# Linked selection, differential introgression and recombination rate variation promote heterogeneous divergence in a pair of yellow croakers

**DOI:** 10.1111/mec.16693

**Published:** 2022-09-29

**Authors:** Le Wang, Shufang Liu, Yang Yang, Zining Meng, Zhimeng Zhuang

**Affiliations:** ^1^ State Key Laboratory of Biocontrol, Institute of Aquatic Economic Animals and the Guangdong Province Key Laboratory for Aquatic Economic Animals, School of Life Sciences Sun Yat‐sen University Guangzhou China; ^2^ Molecular Population Genetics Group, Temasek Life Sciences Laboratory, 1 Research Link National University of Singapore Singapore City Singapore; ^3^ Yellow Sea Fisheries Research Institute, Chinese Academy of Fishery Sciences & Function Laboratory for Marine Biology and Biotechnology Qingdao National Laboratory for Marine Science and Technology Qingdao China; ^4^ Southern Laboratory of Ocean Science and Engineering Zhuhai China

**Keywords:** differential introgression, genomic islands, *Larimichthys*, linked selection, low recombination, secondary contact

## Abstract

Understanding the mechanisms underlying heterogeneous genomic divergence is of particular interest in evolutionary biology. Highly differentiated genomic regions, known as genomic islands, often evolve between diverging lineages. These genomic islands may be related to selection promoting adaptation or reproductive isolation. Based on whole genome assembly and genome‐wide RAD sequencing in a pair of yellow croakers (genus: *Larimichthys*), we investigated the evolutionary processes shaping genomic landscapes of divergence. Demographic modelling indicated that the two species diverged following a secondary contact scenario, where differential introgression and linked selection were suggested to be involved in heterogeneous genomic divergence. We identified reduced recombination rate in genomic islands and a relatively good conservation of both genetic diversity and recombination landscapes between species, which highlight the roles of linked selection and recombination rate variation in promoting heterogeneous divergence in the common ancestral lineage of the two species. In addition, we found a positive correlation between differentiation (*F*_ST_) and absolute sequence divergence (*D*_xy_), and elevated *D*_xy_ in genomic islands, indicating that the genomic landscape of divergence was not shaped by linked selection alone. Restricted gene flow in highly differentiated regions has probably remodelled the landscape of heterogeneous genomic divergence. This study highlights that highly differentiated genomic regions can also arise from a combination of linked selection and differential gene flow in interaction with varying recombination rates.

## 1 INTRODUCTION

Evolutionary mechanisms can shape the genomic architecture that may be related to adaptation and speciation (Cruickshank & Hahn, 2014; Wolf & Ellegren, 2017). Understanding the genomic architecture can help dissect the evolutionary mechanisms and reconstruct the processes between recently evolved species (De La Torre et al., 2019). Recent studies, particularly with the advancement of chromosomal level genome assembly and high throughput sequencing, have uncovered the genomic landscapes of divergence in a wide range of species (Ellegren et al., 2012; Malinsky et al., 2015; Renaut et al., 2013). It has been established that genomic divergence is usually highly heterogeneous across the genome, with some regions being more differentiated (Feder et al., 2012; Wolf & Ellegren, 2017). These regions of elevated genetic differentiation are widely scattered across the whole genome and preferably enriched in some specific regions, thus termed as “genomic islands” (Harr, 2006; Nosil et al., 2009; Turner et al., 2005). Genomic islands were hypothesized to resist gene flow and thus be involved in adaptation and reproductive isolation (Turner et al., 2005). With more and more studies focused on this evolutionary question, a variety of additional evolutionary processes have been discovered to be responsible for such genomic islands (Han et al., 2017; Ravinet et al., 2017; Wolf & Ellegren, 2017). Thus, the potential roles of genomic islands in speciation have been put into controversy (Cruickshank & Hahn, 2014; Nachman & Payseur, 2012; Turner & Hahn, 2010).

Under the hypothesis of speciation‐with‐gene‐flow, genomic islands evolve as speciation islands, as a result of selection acting on beneficial variants (Feder et al., 2012). These regions are expected to spread with divergence time through divergence hitchhiking, while gene flow homogenizes the remaining parts of the genome (Nosil, 2008; Via, 2012). Similarly, genomic islands can also arise in the secondary contact model (Bierne et al., 2013; Harrison & Larson, 2014). Upon secondary contact, genomic differentiation accumulated in allopatry is expected to be homogenized by gene flow in regions that are not involved in reproductive isolation, while the remaining are kept differentiated (Bierne et al., 2013; Harrison & Larson, 2014). However, genomic islands might also evolve as consequences of lineage sorting in the early stage of speciation (Guerrero & Hahn, 2017; Ma et al., 2018), local selection (Tavares et al., 2018) and/or linked selection, such as background selection and selective sweeps in the ancestral lineage and/or acting independently in the divergent populations (Burri, 2017; Burri et al., 2015), regardless of recent gene flow. Especially, when these evolutionary forces interactively act on low‐recombining regions, the effects become more profound (Burri et al., 2015; Ma et al., 2018).

It is challenging to answer whether genomic islands indicate the existence of local genetic barriers to gene flow. First, because in the absence of gene flow or under limited gene flow, selection mechanisms that are not directly related to speciation can cause variation in the rate of lineage sorting along the genome, and hence, incidental islands of *F*_ST_ in low‐recombining regions arise through linked selection (Burri et al., 2015; Cruickshank & Hahn, 2014; Vijay et al., 2017). These are usually associated with “valleys” of absolute divergence (*D*_xy_), because ancestral variation was also probably reduced in low‐recombining regions in the ancestral population (Burri et al., 2015; Cruickshank & Hahn, 2014). Second, in the presence of substantial gene flow, genomic islands may have formed due to the effect of local selection in the face of gene flow (i.e. primary divergence), or alternatively due to differential erosion of divergence during secondary contact (Cruickshank & Hahn, 2014; Han et al., 2017; Wolf & Ellegren, 2017). However, *D*_xy_ is expected to be increased in both primary divergence with gene flow and differential introgression after secondary contact, but reduced in pure allopatric (or post‐speciation) divergence because of linked selection removing diversity in low recombining regions in the ancestral population even before divergence starts (Burri et al., 2015; Cruickshank & Hahn, 2014). Therefore, it is necessary to determine the conditions under which genomic islands have formed with respect to gene flow (i.e., no gene flow, continuous gene flow or secondary contact). Under the secondary contact scenario, the overall heterogeneous divergence pattern may result from a mix of heterogeneous divergence during the allopatric phase due to linked selection, followed by heterogeneous introgression during secondary contact due to the presence of genetic barriers (i.e., local adaptations or genetic incompatibilities) that locally reduce gene flow.

The small and large yellow croakers (*Larimichthys polyactis* and *L. crocea*) are the only two sister species within the genus *Larimichthys* of family Sciaenidae. The two species are critically important fishery resources endemic to the Northwest Pacific. *L. polyactis* distribute from the East China Sea northward to the Bohai Bay, while *L. crocea* from the South China Sea northward to the East China Sea, with their distribution ranges overlapping in the East China Sea (Wang, Liu, et al., 2013; Wang, Shi, et al., 2013). The two species differ in body size, spawning migration routes and overwintering aggregation grounds, but slightly overlap in spawning time and spawning grounds and nursery areas in the northern East China Sea (Ikeda, 1964; Liu, 1964). Previous studies have suggested that *L. polyactis* and *L. crocea* were isolated mainly in two different refuges during Pleistocene glacial times, located in the East and South China Sea, respectively (Wang, Shi, et al., 2013; Xiao et al., 2009). Following recent glacial retreat, both species have experienced rapid expansions and partial contact, and adapted to diversified environmental conditions, for example, spawning temperature and salinity (Liu et al., 2016; Wang, Liu, et al., 2013; Wang, Shi, et al., 2013). In the overlapping distribution region, we observed population mixture of the two species, implying recent interspecific gene flow although we could not exclude the possibility that there was absence of gene flow (Wang et al., 2012). Hence, this system might follow a speciation model of secondary contact. Therefore, the two species provide a valuable system for examining how differential evolutionary factors including gene flow, genetic barriers, selection and recombination landscapes, could have shaped the patterns of genomic divergence in the mode of secondary contact.

Here, we first sequenced the genome of *L. polyactis* and obtained genome‐wide variants of the two sister species in their distribution range, by RAD (restriction‐site associated DNA) sequencing. We then examined the demographic history of the two species taking into consideration both heterogeneous introgression and linked selection in the genome, and tested whether the divergence conforms to the secondary contact scenario. Under this scenario, we expected restricted gene flow in genomic islands in comparison to the rest of the genome. In addition, we analysed the genome‐wide patterns of genetic diversity to examine the hypothesis of “genomic islands” for interspecific divergence. In particular, by comparing the genetic diversity measure between genomic islands and the remaining regions, we infer the roles of gene flow, selection and recombination in promoting heterogeneous genomic divergence during two successive phases of speciation. Furthermore, by comparing the genomic patterns between interspecific and intraspecific divergence, we tested if the mechanisms that influence the genomic patterns of interspecific divergence also affect that of intraspecific differentiation. This study provides not only valuable genomic resources for the two sister species, but also novel insights into the evolutionary factors underlying genomic islands in the studied system.

## 2 MATERIALS AND METHODS

### 2.1 Ethics statement

The studied species are not protected by Chinese law or by any of the countries where the sampling was performed. It is a commercially harvested species in Northeast Asian countries. The samples were collected by trawling at the designed sampling sites and were already dead when sampling.

### 2.2 Samples for genome, transcriptome and RAD sequencing

One female *L. polyactis* was selected for genome sequencing. One 20‐kb library was constructed and sequenced on PacBio Sequel II (Pacific Biosciences) by BGI (Shenzhen). Both paired‐end (PE) (270 and 550 bp) and mate pair (MP) (5 and 10 kb) libraries were also constructed from the same sample (Table S1). Three mRNA libraries were separately constructed for three individuals (two females and one male at 1 year old), by pooling total RNA from multiple tissues: brain, eye, gill, intestine, spleen, liver, heart, kidney, gonad, skin and muscle, with equal amounts from each tissue. PE, MP and mRNA libraries were sequenced for 2 × 150 bp by BGI (Shenzhen), using Illumina HiSeq 4000 (Illumina).

**FIGURE 1:**
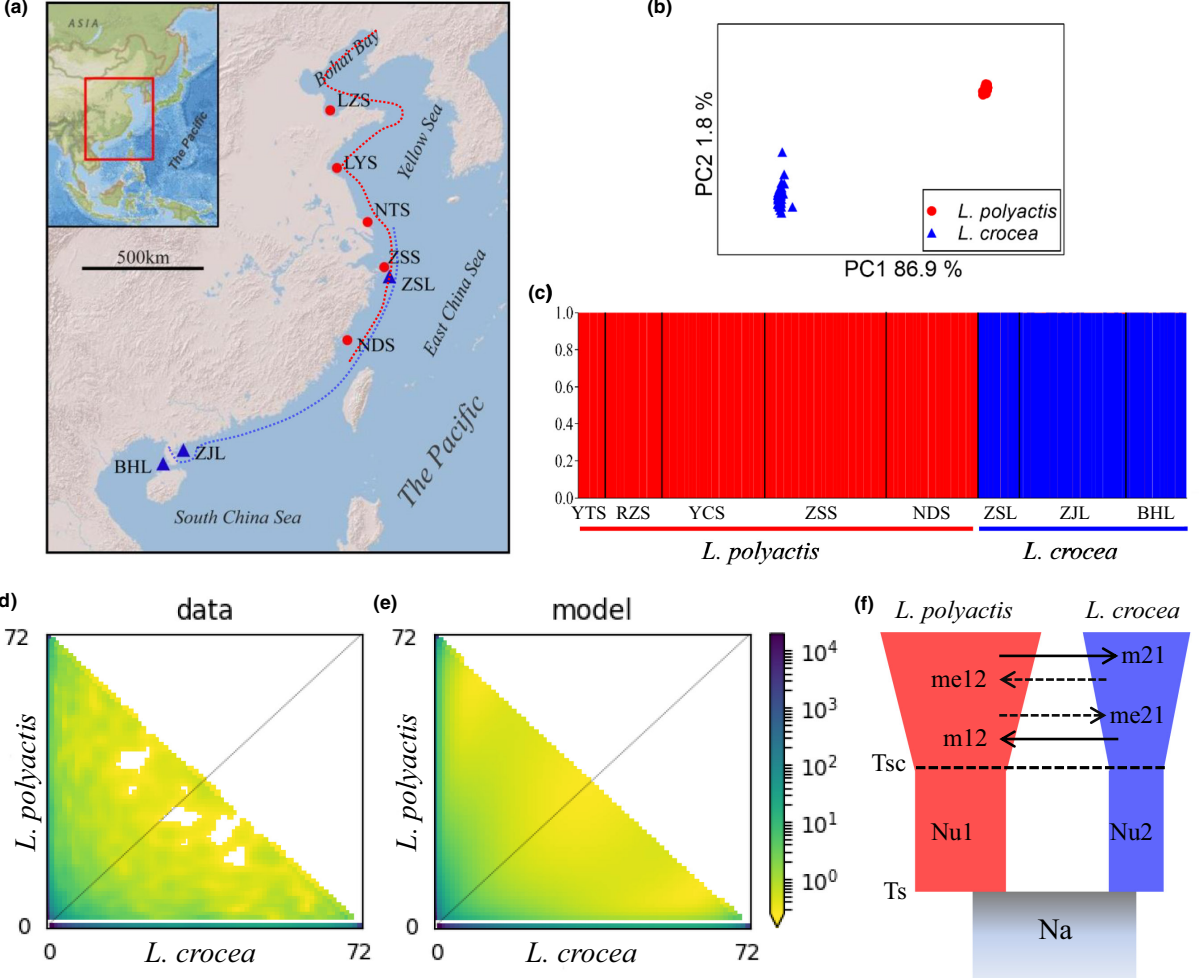
Population structure and demographic history between *L. polyactis* and *L. crocea*. (a) Sampling locations of *L. polyactis* (five, red circles) and *L. crocea* (three, blue triangles) along coastal waters of the Northwest Pacific, where the distribution range of *L. polyactis* and *L. crocea* are indicated with red and blue dashed lines, respectively. (b) Principal component analysis (PCA) of population structure between *L. polyactis* and *L. crocea*, based on common SNP data set. (c) Genetic clusters showing population structure between *L. polyactis* and *L. crocea*, based on common SNP data; (d) and (e) showing the folded joint site frequency spectrum (SFS) and the expected folded joint SFS under the most fitting demographic scenario (SC2N2mG), respectively, between *L. polyactis* and *L. crocea*. (f) The SC2N2mG demographic scenario between *L. polyactis* and *L. crocea*. Na indicates effective population size of ancestral population; Nu1 and Nu2 indicate the effective population size of the *L*. *polyactis* and *L. crocea* lineages after split from their common ancestral population, respectively; m12 and m21 indicate the homozygous gene flow, while me12 and me21 indicate heterozygous gene flow from one species to the counterpart; T_S_ denotes the split time of ancestral population and T_SC_ is the time when ancient isolation stops.

One hundred and five *L. polyactis* from five locations and 55 *L. crocea* from three locations were collected across their distribution ranges (Table S2 and Figure 1a). DNA was isolated from fin using standard phenol‐chloroform extraction protocol. RAD libraries of 500‐bp inserts were constructed according to a previous protocol (Baird et al., 2008), using restriction enzyme PstI‐HF (New England Biolabs). The libraries were sequenced on Illumina HiSeq 3000 platform (Illumina) in single end with read length of 150 bp.

### 2.3 Genome assembly and annotation

Both PE (~55×) and MP reads (~ 40×) were first cleaned using process_shortreads in Stacks version 1.45 (Catchen et al., 2011). ALLPATHS‐LG version 52,488 (Gnerre et al., 2011) was then used to estimate genome size based on k‐mer frequencies and de novo assemble the genome. PacBio reads of ~80× coverage were assembled using Canu version 1.8 (Koren et al., 2017), with an estimated genome size of 650 Mb (Figure S1). The assembly was then polished with Pilon version 1.22 (Walker et al., 2014), using both cleaned PE and MP libraries. There are no genomic resources available for chromosome‐level genome assembly of *L. polyactis*. Due to a recent divergence at <5 million years ago (Ma) (Liu et al., 2010), *L. polyactis* and *L. crocea* probably have a high level of genome synteny. Comparison of long scaffolds between the two species found no evidence of chromosome rearrangements, except for six small‐scale inversions (see below). We therefore anchored the scaffolds of *L. polyactis* to the high‐density genetic maps of *L. crocea* (24 linkage groups [LGs], 2.7 cM/Mb and an average marker resolution of 0.36 cM) (Kong et al., 2019) to achieve a chromosome‐level assembly using ALLMAPs (Tang et al., 2015) with default parameters.

Completeness of genome was assessed by BUSCO version 3.0.1 (Simão et al., 2015). MAKER2 (Cantarel et al., 2008) was used to annotate the genome according to the description in Appendix S1. We further predicted conserved noncoding elements (CNEs), according to a previous method (Brawand et al., 2014) (Appendix S1).

### 2.4 Comparative genomic analysis between *L. polyactis* and *L. crocea*

Genomic synteny between *L. polyactis* and stickleback (Jones et al., 2012) was studied based on one‐to‐one orthologous genes, which were identified by pairwise blast search using Orthologue‐finder (Horiike et al., 2016) based on OrthoMCL database version 5 (Chen et al., 2006). We further identified chromosome rearrangements between the two sister species. Since the pseudochromosome sequences of *L. polyactis* were dependent on the genetic maps of *L. crocea*, we could not examine rearrangements by pairwise chromosome alignment. Instead, we screened fragment rearrangements by pairwise alignment of scaffolds, which were directly assembled using PacBio reads for both species (Chen et al., 2019). Putative rearrangements were then verified by examining the linkage and/or recombination rates around the breaking points. If there was a fragment rearrangement in one species, for example, *L. polyactis*, markers flanking the breakpoints would be in tight linkage when using the genomic coordinates of *L. polyactis* for the markers. In contrast, the linkage between markers flanking the breakpoint would be distorted when using the genomic coordinates of *L. crocea* for the markers.

### 2.5 Genome‐wide SNP discovery and genotyping

Raw RAD sequencing reads were filtered using process_radtags (−r ‐c ‐q ‐t 135) in Stacks version 1.45 (Catchen et al., 2011). An average number of 6.2 M clean reads was obtained per sample. We found that 98.6% and 98.2% of reads of *L. polyactis* and *L. crocea*, respectively, were mapped to the genome of *L. polyactis*, while 96.9% and 98.4% of reads of *L. polyactis* and *L. crocea*, respectively, were mapped to the genome sequences of *L. crocea*. The genome sequence of *L. polyactis* was used as reference due to the slightly higher mapping rates. Clean reads were aligned to the reference genome using BWA‐MEM (Li & Durbin, 2009) with default parameters. Uniquely aligned reads were used for SNPs discovery and genotyping using Stacks version 1.45 (Catchen et al., 2011) with default parameters. We further filtered out the variants that had more than two alleles, deviated from Hardy–Weinberg equilibrium (HWE) in a single population at the significance threshold of 0.001 and showed heterozygosity of >0.5. Only SNPs that were present in >70% of individuals in each location, were retained for further analysis.

### 2.6 Genetic diversity and population structure

Genetic diversity in terms of nucleotide diversity (π) was estimated by taking monomorphic sites into consideration using VCFtools version 0.1.15 (Danecek et al., 2011). The program ARLEQUIN version 3.5 (Excoffier & Lischer, 2010) was used to estimate pairwise *F*_ST_ among locations and examine the significance of differentiation using Fisher's exact test. Principal components analysis between and within species, was performed using Plink2 (Purcell et al., 2007). Population structure in the form of genetic clusters was analysed using Admixture version 1.3.0 (Alexander & Lange, 2011), with the number of genetic clusters ranging from 2 to 10. For these analyses, we filtered the SNPs that show high linkage disequilibrium: only one SNP was retained from each RAD tag and those with neighbouring SNPs on different RAD tags and showing elevated LD (*R* ^2^ > 0.1) within 100 kb window were excluded. Patterns of isolation‐by‐distance were examined using Mantel test (Smouse et al., 1986).

### 2.7 Modelling demographic history

The demographic history of divergence between species was studied by modelling the alternative two‐population demographic scenarios based on joint site frequency spectrum (SFS), with the program DaDi version 1.6.3 (Gutenkunst et al., 2009). Due to very weak intraspecific differentiation (*F*_ST_ <0.01), we pooled samples separately for each species. We selected 45 individuals separately from *L. polyactis* and *L. crocea* that had the least missing data. With missing data of <10%, 86,854 SNPs were retained to generate the folded joint SFS. We further projected down the folded SFS to a phase, in order to minimize the effect of missing data and meanwhile maximize the effective number of samples. We first fitted the four standard alternative scenarios of historical divergence according to our previous study (Wang et al., 2016), including the strict isolation model (SI), isolation‐with‐migration model (IM), ancient migration model (AM) and secondary contact model (SC), where the gene flow was assumed to be shared among loci. We further fitted the folded SFS to three modified models: IM2m, AM2m and SC2m, corresponding to the above IM, AM and SC models, respectively. The modified models allow heterogeneous gene flow across the genome using two categories of effective migration rates among loci (−2 m) (Tine et al., 2014). Three additional modified alternative scenarios: IM2N, AM2N and SC2N that take into consideration the Hill–Robertson effect (Hill & Robertson, 1966) or effect of linked selection, were further modelled to fit the spectrum by considering two categories of loci (‐2 N) occurring in the genome (Rougeux et al., 2017). We also modified the four standard alternative models by allowing population expansion (−G) (SIG, IMG, AMG and SCG). The best fitting models within each category (standard, −2 m, ‐2 N and ‐G), were inferred from Akaike information criterion (AIC), and were then optimized by simulation of historical population growth, allowing heterogeneous gene flow and/or considering the effect of linked selection (−2mG, ‐2NG and ‐2N2mG), to capture additional residuals of the folded SFS. The parameter estimates from each alternative scenario were examined for convergence by running the program for 20 replicates with each using independent starting parameters, and the confidence intervals of each parameter for the best fit model were obtained by bootstrapping over loci for 100 times.

### 2.8 Detection of outlier loci influenced by selection

Footprints of local selection within each species were first detected using FDIST2 (Beaumont & Balding, 2004), which assumes that loci under putative diversifying selection would show elevated population differential condition on their heterozygosity. Loci that were distributed above 99% quantiles of the null distribution and were significant at 0.01 level after FDR corrections were considered as outliers. We further employed a Bayesian simulation‐based test to identify signatures of local selection using BAYESCAN version 2.1 (Foll & Gaggiotti, 2008), which works by decomposing locus‐specific *F*_ST_ into a population and a locus specific component, and evaluates the necessity to include the locus specific component to account for selection. Posterior odds (PO) were estimated for each locus and loci that were significant at 0.01 level were considered as outliers. For these analyses, only SNPs with minor allele frequency >0.05 were used, in order to simulate the null distribution and reduce false positives in the background of high gene flow. Outlier SNPs that were located in the genomic regions of protein coding genes and within its 1‐kb flanking regions were considered for functional enrichment analysis. Genes under positive selection and related to outliers were extracted from genome annotation. GO IDs of these genes were obtained by blast search against the zebrafish protein database (Ensembl database release 96) using blastp with an E‐value cutoff <1E‐10. Gene ontology (GO) enrichment analysis was carried out by comparing the GO IDs of the outlier genes with that of all genes of zebrafish, using the program Metascape (Zhou et al., 2019) with default parameters.

### 2.9 Identifying genomic regions of elevated differentiation

In order to identify genomic islands, we partitioned the genome sequences into 150‐kb nonoverlapping windows and estimated both interspecific and intraspecific *F*_ST_ for each window. Unweighted *F*_ST_ was estimated with VCFtools version 0.1.15 (Danecek et al., 2011). Windows of less than five SNPs were removed, which kept 3706 windows for further analysis. We estimated the distribution of *F*_ST_ across windows and those within the upper 5% quantile of the *F*_ST_ value distribution were considered as genomic islands, according to previous studies (Le Moan et al., 2021; Liu et al., 2020). Adjacent outlier windows were merged as one. We compared the other window sizes of 100, 200 and 500 kb to infer evolutionary mechanisms and found little difference among the results. We also selected windows within the upper 1% quantile of the *F*_ST_ value distribution, which did not lead to different results with the windows within the upper 5% quantile. For intraspecific divergence, 3411 windows were retained for inferring genomic islands. Intraspecific genomic islands were defined using the same criterion as the above. Intraspecific *F*_ST_ was estimated across all populations using hierarchical island model with the R package “hierfstat” (Goudet, 2005).

### 2.10 Inferring factors shaping heterogeneous genomic divergence

To dissect the factors promoting heterogeneous genomic divergence, we estimated the genetic diversity parameters of genomic islands in comparison to the rest of the genome. Absolute sequence divergence, *D*_xy_, between *L. polyactis* and *L. crocea*, was calculated for individual windows, using a perl script (Han et al., 2017). Window‐sized estimation of nucleotide diversity (π) and Tajima's *D* were calculated using VCFtools version 0.1.15 (Danecek et al., 2011). Tajima's *D* is often used to detect selective sweeps. Negative Tajima's D values indicate an excess of low frequency polymorphisms relative to equilibrium expectation, indicating recent selective sweep or population expansion after recent bottleneck. Population‐scaled recombination rate along individual chromosomes, was measured as ρ = 4Ner per kb and estimated using LDhat version 2.1 (McVean et al., 2004) with 150‐kb nonoverlapping sliding windows, with parameters: ‐bpen 10, −its 10,000,000, −theta 0.05. The differences of genetic diversity measures between genomic islands and the rest of the genome were examined using Mann–Whitney U test. The correlations of genomic landscapes between genetic diversity measures were assessed using Pearson's correlation coefficient.

## 3 RESULTS

### 3.1 Genome assembly and annotation

Genome assembly of *L. polyactis* was ~653 Mb, with contig N50 of 2.47 Mb. Approximately 95.7% of total sequences were anchored onto high density linkage maps of *L. crocea*, with N50 length of pseudomolecules of 37.31 Mb (Table S3). Assessment of genome completeness showed that only 1.7 and 0.% of the BUSCO genes, respectively, were missing and fragmented, indicating the high quality of the genome assembly (Table S4). Repeated sequences accounted for ~21.1% of the genome, in which 83.7% were transposable elements (Table S5). A total of 24,625 protein coding genes were predicted in *L. polyactis*, showing little differences with that of *L. crocea* (23,657 and 25,401 for two different assemblies, respectively) (Ao et al., 2015; Wu et al., 2014). We predicted 235,237 CNEs with an average length of 202 bp.

Overall GC content in the genome of *L. polyactis* was 42.3%. However, it was not randomly distributed throughout the genome. For some chromosomes, for example, chr1 and chr24, GC content was higher towards both ends, while for some other chromosomes, for example, chr4 and chr5, GC rich sequences were located in the middle of the chromosomes (Figure S2). In addition to GC content, repeated sequences, recombination rates, as well as CNEs were also observed not to be randomly distributed across the whole genome (Figure S2). Interestingly, we found that GC content was positively correlated with recombination rates (*R* = 0.596, *p* < 10^−15^) and content of repeated sequences (*R* = 0.435, *p* < 10^−15^), but negatively correlated with content of CNEs (*R* = −0.309, *p* < 10^−15^). Further investigation showed that content of repeated sequences was also positively and negatively correlated with recombination rates and content of CNEs, respectively (Figure S3).

We compared the genomic synteny between *L. polyactis* and stickleback. A high collinearity was observed between any pair of homologous chromosomes (Figure S4), except for some evidence of genome rearrangements (Figure S5), although the two species have diverged since ~100 million years ago (http://www.timetree.org/). Six putative chromosome inversions were identified between *L. polyactis* and *L. crocea*, at contig level (Figure S6). With sufficient marker resolution, we estimated the recombination rates around the inverted region of the longest fragment inversion. We found that recombination rates in the regions closely flanking the breaking points were increased for *L. crocea* and *L. polyactis* when referenced to the genome of *L. polyactis* and *L. crocea*, respectively (Figure S7). Such distortion of recombination rates around the breaking points was suggested as a feature of chromosome inversion (Crown et al., 2018).

### 3.2 Population structure between and within species

After quality control, a total number of 1,113,736 SNPs were obtained. The SNPs were distributed on 88,300 RAD tags throughout the two sister species, within which 149,201 SNPs distributed on 61,346 RAD tags showed a minor allele frequency of ≥0.05 (Table S6). Within each species, 806,471 and 537,052 SNPs distributed on 87,701 and 83,264 RAD tags were obtained, for *L. polyactis* and *L. crocea*, respectively. A total of 129,768 and 114,520 SNPs, distributed on 54,059 and 48,672 RAD tags showed a minor allele frequency of ≥0.05 for *L. polyactis* and *L. crocea*, respectively (Table S6).

Overall genetic diversity in terms of nucleotide diversity (π) showed little difference between species (*L. polyactis*: 0.0048–0.0056 vs. *L.crocea*: 0.0040–0.0050) and among locations within species (Table S2). We observed clear divergence between species (*F*_ST_, ~0.6), but very weak population differentiation within each species, with *F*_ST_ ranging from 0.0001 to 0.0068 and from 0.0009 to 0.0014 for *L. polyactis* and *L. crocea*, respectively (Table S7). In particular for *L. polyactis*, none of the pairwise *F*_ST_ across all studied locations was significant except for one pair between the northernmost (LZS) and the southernmost (NDS) locations (Table S7). Overall population differentiation for *L. polyactis* did not follow a pattern of isolation‐by‐distance (Mantel test, *R* = 0.408, *p* > .05). Consistently, both PCA and Admixture analysis revealed striking interspecific genetic divergence (Figure 1b,c). However, we did not observe a significant geographical pattern of divergence within each species (Figures S8–S11).

### 3.3 Demographic history of speciation

A total of 17 alternative demographic models were designed to fit the folded joint SFS (Figure S12). Among the four standard alternative models, SC was indicated to have the best support, showing the highest maximum‐likelihood value and lowest AIC score (Table S8). In agreement with the above results, SC2m and SC2N were the best supported scenarios in the two categories of custom alternative scenarios that take into consideration the heterogeneous migration (−2 m) and the effect of linked selection (‐2 N) in the genome, respectively (Table S8). Among the four modified alternative models that allow population expansion (−G), SCG was observed as the best model to capture the folded spectrum. Finally, we modified the SC model by allowing population expansion, heterogeneous migration and/or linked selection (SC2mG, SC2NG and SC2N2mG) and observed substantial improvement for these models to capture the folded spectrum. SC2N2mG was the best supported model among all examined alternative scenarios (Figure 1d–f and Table S8), where the folded SFS was fairly well captured and most of the residuals were in a very narrow range from −1 to 1 (Figure S13).

We converted the demographic parameters in units of 2Na generations into biologically meaningful units using the formula Na = theta/(4*μ*L), where μ is the mutation rate fixed at 10^−8^ mutations per site per generation and L is the effective length of the genome explored by RAD sequencing (Rougeux et al., 2017). Under SC2N2mG, ~16.3% of genome was observed to have a reduction in effective migration rates compared to the rest of the genome (Table S9). The effective migration rates of genomic‐island genes from *L. polyactis* to *L. crocea* (1.851E‐07) and from *L. crocea* to *L. polyactis* (1.481E‐07) were more than 10 times lower than those for neutral migration rates (3.701E‐06 and 5.120E‐06, respectively) (Table S9). The proportion of the genome with a locally reduced effective size due to the effect of background selection and selective sweeps was estimated to be ~95.4%, but the reduction in effective size in these regions was estimated to be only ~6.9% (Table S9). Using an average generation time of 3 years (Liu, 1962), the divergence time between *L. polyactis* and *L. crocea* was dated to ~1.19 (95% CI: 1.10–1.28) Ma. The time when the two species had secondary contact was estimated to be relatively ancient near ~0.81 (95% CI: 0.77–0.86) Ma. The population sizes for *L. polyactis* and *L. crocea* were revealed to have dramatically expanded by ~10 and ~110 times, respectively.

### 3.4 Adaptive divergence within species

Outlier tests identified 779 and 206 SNPs under putative local selection for *L. polyactis* and *L. crocea*, respectively (Table S10). Only two outliers were overlapping between species. Annotation against the reference genome revealed that 457 (58.7%) and 125 (60.7%) outliers were associated with 440 and 125 unique protein coding genes for *L. polyactis* and *L. crocea*, respectively, which was higher than the ratio under the expectation of random distribution that ~45% of outliers are located in genic regions. We further observed that 23.2% (181) and 27.7% (57) of outliers were located in coding sequences for *L. polyactis* and *L. crocea*, respectively, which were also higher than the ratio under the expectation of random distribution that ~7% of outliers hit the coding sequences in the genome. Only 10 outlier genes were overlapping between species. Gene ontology analysis showed that there was no overlapping for the enriched terms between *L. polyactis* and *L. crocea* (Figure S14).

**FIGURE 2:**
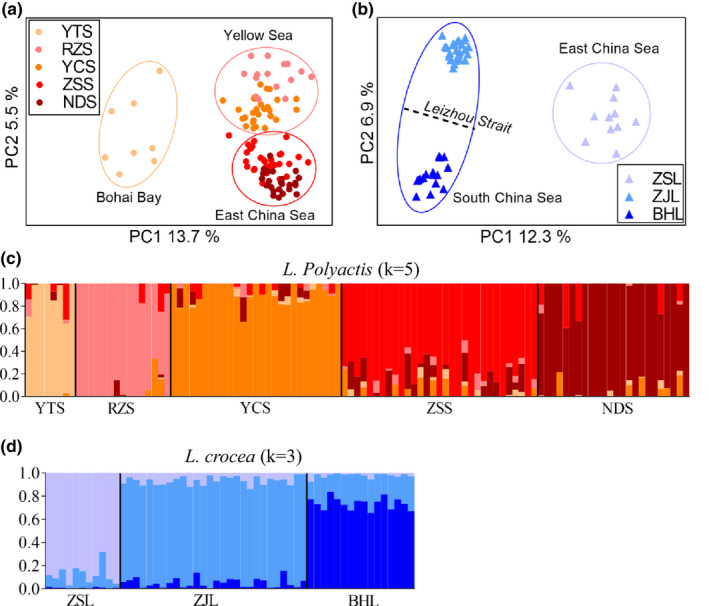
Adaptive population differentiation within *L. polyactis* and *L. crocea*. (a) Population structure revealed by PCA among five locations of *L. polyactis*, based on outlier loci influenced by putative local selection, where samples from the Bohai Sea, the Yellow Sea and the East China Sea are highlighted with circles. (b) Population structure revealed by PCA among three locations of *L. crocea*, based on outlier loci influenced by putative local selection, where samples from the each China Sea and the South China Sea are highlighted with circles. (c) Genetic clusters inferred by Admixture among five locations of *L. polyactis* at the most likely K value of 5, based on outlier loci, where the colours of genetic clusters correspond to those of locations in (a), and (d) genetic clusters among three locations of *L. crocea* at the most likely K value of 3, based on outlier loci under putative local selection, where the colours of genetic clusters are corresponding to those of locations in (b).

Population differentiation based on outliers were statistically significant and much higher than that of neutral differentiation (*F*_ST_ ranging from 0.03 to 0.28 and from 0.07 to 0.14 for *L. polyactis* and *L. crocea*, respectively), and presented clear geographical patterns, for both species (Table S11–12). PCA analysis showed that *L. polyactics* were differentiated into three populations, corresponding to the Bohai Bay, the Yellow Sea and the East China Sea, respectively, while *L. crocea* were also clustered into three populations: one population in the East China Sea and two populations in the South China Sea that were separated by the Leizhou Strait (Figure 2a,b). Admixture analysis revealed that five locations of *L. polyactis* and three locations of *L. crocea* were distinctively differentiated with each other within species (Figures 2c,d, S15 and 16). Interestingly, the overall population differentiation at outlier loci presented a significant pattern of isolation‐by‐distance for *L. polyactis* (Mantel test, *R* = 0.738, *p* < .05), contradicting with that of neutral differentiation (Figure S17).

### 3.5 Heterogeneous genomic divergence between species

**FIGURE 3:**
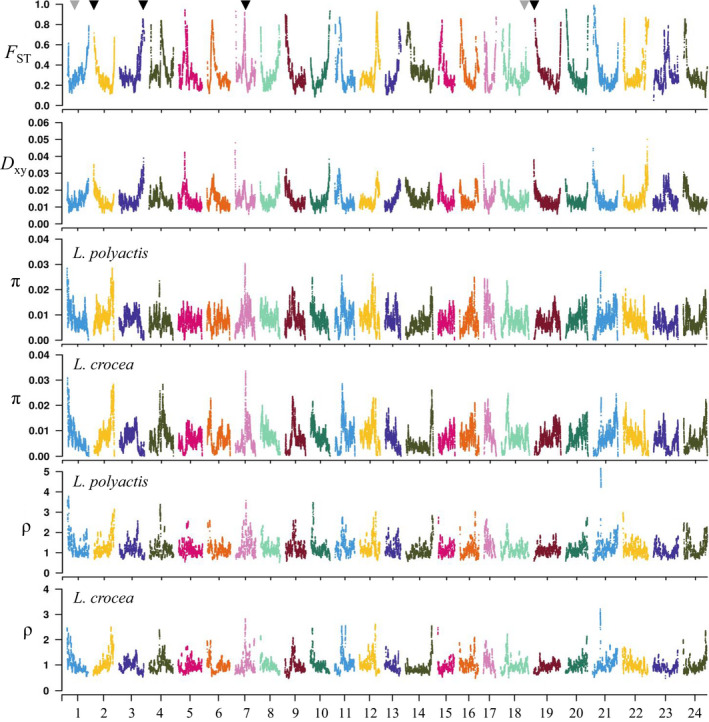
Genome‐wide heterogeneous divergence between and within *L. polyactis* and *L. crocea*. Genome‐wide patterns of interspecific divergence (*F*_ST_) and absolute sequence divergence (*D*_xy_) between *L. polyactis* and *L. crocea*, intraspecific nucleotide diversity (π) and recombination rate (ρ) separately for each sister species *L. polyactis* and *L. crocea*, were estimated along individual chromosomes based on 150 kb‐windows. Six putative chromosomal inversions are indicated with triangles, among which four overlapped with genomic islands at chr2, chr3, chr7 and chr19, respectively, are highlighted with black.

We identified 80 regions with elevated differentiation (*F*_ST_, 0.947 ± 0.020) in comparison to the rest of the genome (*F*_ST_, 0.627 ± 0.148; Mann–Whitney U test*, p* < 10^−15^). These genomic islands were distributed across almost all chromosomes, with most of the peaks emerging towards the two ends of the chromosomes while the others were in the middle of the chromosomes (Figure 3). Interestingly, out of the six putative chromosomal inversions identified between species, four located in the genomic islands at chr2, chr3, chr7 and chr19, respectively (Figure 3). These genomic islands showed significantly reduced nucleotide diversity (π) and skews towards an excess of rare alleles (as examined in terms of reduced Tajima's *D*) in each species (Table S13).

To explore what factors have contributed to the heterogeneous genomic divergence, we examined the distribution pattern of *D*_xy_ throughout the genome. Consistent with the patterns of *F*_ST_, *D*_xy_ was also observed to be elevated in genomic islands where gene flow was restricted as revealed by demographic history modelling, in comparison to the rest of the genome (*D*_xy_, 0.030 vs. 0.016; Mann–Whitney *U* test, *p* < 10^−15^). In addition, *D*_xy_ showed a genome‐wide positive correlation with *F*_ST_ (Pearson's correlation: *R* = 0.463, *p* < 10^−15^) (Figure S18).

**FIGURE 4:**
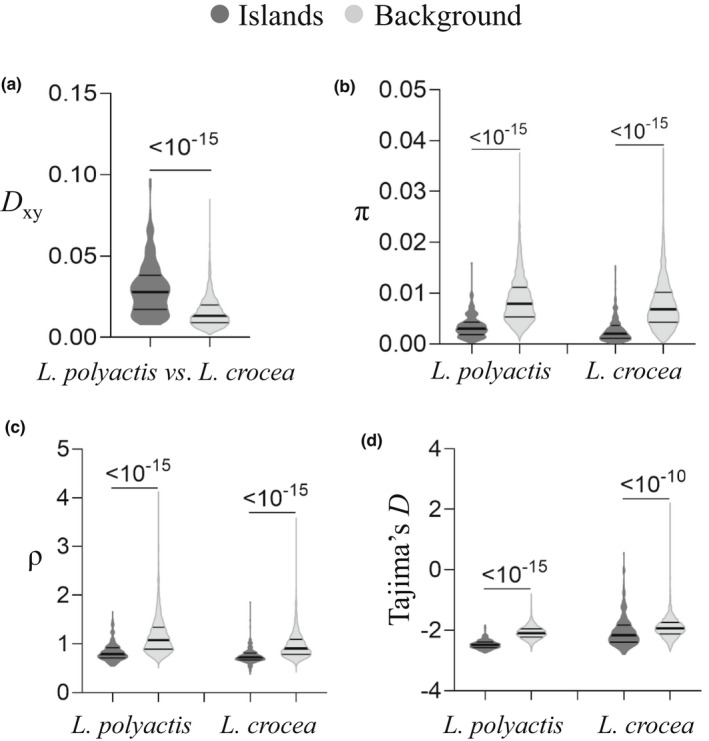
Genome‐wide heterogeneous divergence between *L. polyactis* and *L. crocea*. (a–d) show the comparisons of absolute sequence divergence (*D*_xy_), nucleotide diversity (π), recombination rate (ρ) and Tajima's *D* between genomic islands and the rest of the genome for interspecific divergence between *L. polyactis* and *L. crocea*, respectively, where *p*‐values for Mann–Whitney U tests are indicated above the plots.

**FIGURE 5:**
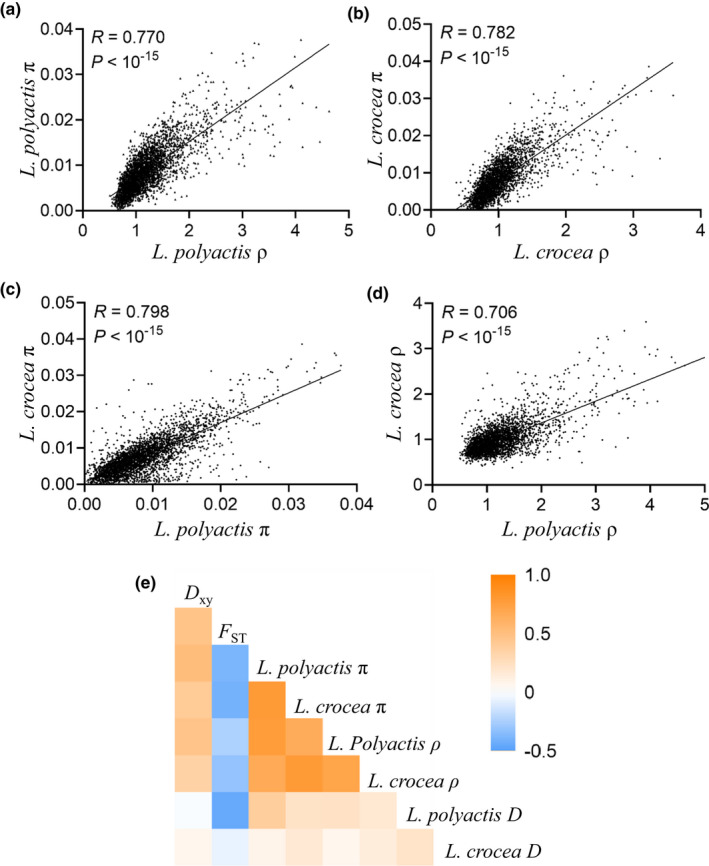
Pairwise correlations between genetic diversity measures between and within species. (a) and (b) show the overall Pearson's correlation between recombination rate and nucleotide diversity for *L. polyactis* and *L. crocea*, respectively. (c) and (d) show the overall Pearson's correlation of nucleotide diversity and recombination rate, respectively, between *L. polyactis* and *L. crocea*. (e) a heatmap showing the overall Pearson's correlation among *D*_xy_, *F*_ST_, π, ρ and Tajima's *D* between and within *L. polyactis* and *L. crocea*.

To uncover additional factors leading to heterogeneous divergence, we further examined the distribution pattern of recombination rate (ρ) throughout the genome. In agreement with the distribution pattern of π, we observed that ρ was also significantly reduced in genomic islands compared to the rest of the genome in both species (Figure 4; Table S13). Interestingly, we observed that π and ρ were not only positively correlated with each other within each species (Pearson's correlation: *R* = 0.770, *p* < 10^−15^ and *R* = 0.782, *p* < 10^−15^ for *L. polyactis* and *L. crocea*, respectively; Figure 5a,b), but also separately positively correlated between *L. polyacits* and *L. crocea* (π: *R* = 0.798, *p* < 10^−15^ and ρ: *R* = 0.706, *p* < 10^−15^) (Figure 5c,d), supporting a relatively good conservation of both nucleotide diversity and recombination landscapes between species. The overall distribution of π and ρ across chromosomes presented negative relationships with *F*_ST_ for both species (Figures 5e and S19). These observations suggest that linked selection in low recombining regions including genomic islands played crucial roles in the ancestral lineage before divergence of the two species. Interestingly, we found a global pattern of positive correlations between π and *D*_xy_, and between ρ and *D*_xy_, for both species (Figure 5e).

### 3.6 Intraspecific versus interspecific heterogeneous divergence

**FIGURE 6:**
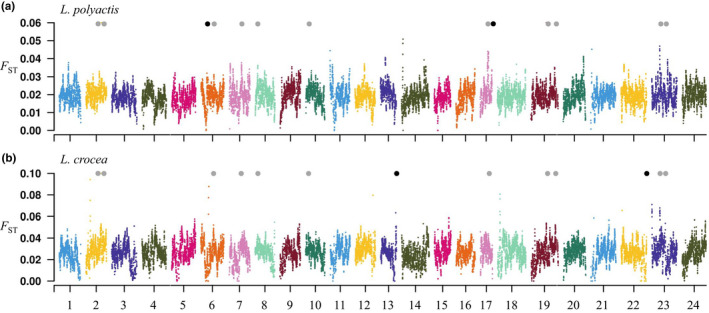
Genome‐wide patterns of heterogeneous divergence within *L. polyactis* and *L. crocea*. (a) and (b) genome‐wide patterns of genetic divergence within *L. polyactis* and *L. crocea*, respectively, estimated based on 150‐kb sliding windows. Overlapping genomic islands between the two species are indicated with grey solid dots, while overlapping genomic islands between intraspecific and interspecific divergence are highlighted with black solid dots.

In high gene flow background, we observed evidently elevated genomic regions across most of the chromosomes for both species, although the genomic islands were much less pronounced than those of interspecific divergence (Figure 6a,b). A total of 144 and 142 genomic islands were identified in *L. polyactis* and *L. crocea*, respectively. These genomic islands showed a slight reduction in both nucleotide diversity and recombination rate in comparison to the rest of the genome in *L. polyactis*, but not in *L. crocea* (Table S14). We identified 11 (~8%) intraspecific “genomic island” overlapping between species (Figure 6a,b). There was a weak positive correlation for the distribution of lineage‐specific *F*_ST_, between *L. polyactis* and *L. crocea* (Pearson's correlation: *R* = 0.135, *p* < 10^−14^). Interestingly, we observed that 5.0% (4) of the interspecific “genomic islands” coincided with intraspecific ones for either *L. polyactis* (2) or *L. crocea* (2) (Figure 6a,b), slightly lower than that of the expectation under random distribution of ~7.3%. We mapped outlier loci putatively influenced by local selection in either species to the genome and found that these loci were not enriched in interspecific genomic islands. Only 12 out of 984 (1.2%) were located in these regions, less than that of the expectation under random distribution of 4.2%. Interestingly, we observed significantly negative correlations between interspecific *F*_ST_ and intraspecific *F*_ST_ for both species (Pearson's correlation: *R* = −0.312, *p* < 10^−15^ and *R* = −0.299, *p* < 10^−15^ for *L. polyactis* and *L. crocea*, respectively).

## 4 DISCUSSION

In this study, we assembled a chromosome‐level genome sequence of *L. polyactis*. Based on the genome, we modelled the demographic history and analysed the genome‐wide patterns of genetic diversity between and within two sister species: *L. polyactis* and *L. crocea*. Our data support that the two species diverged following a secondary contact scenario, and the genome‐wide heterogeneous divergence between species is due to linked selection in the two species and restricted gene flow in highly differentiated regions in the phase of secondary contact. Our results also address the crucial roles of recombination rate variation in facilitating evolution of highly differentiated regions throughout the whole genome.

### 4.1 Important genomic resources for *Larimichthys*

In this study, we assembled and annotated a high‐quality genome sequence of *L. polyactis*. In particular, beyond protein coding genes, we predicted 235,237 CNEs throughout the whole genome. Previous studies have shown that CNEs can affect the expression of neighbouring genes by cis‐regulation (Wang et al., 2022, 2021). Therefore, sequence variations in CNEs could also play important roles in bringing about phenotypic innovations. The chromosomal‐level genome sequence provides valuable resources for investigation of the genome‐wide landscape of divergence. We detected very low genetic differentiation within each species using genome‐wide variants, indicating high gene flow. In particular, *L. polyactis* was shown to be nearly panmictic in the distribution range. However, a lack of geographical pattern of population differentiation notably affects management and conservation of the natural resources of both species (Wang et al., 2015). Outlier loci can increase the power of mixed stock analysis between migratory groups of *L. polyactis* (Wang et al., 2015). Here, we separately identified hundreds of outlier loci putatively influenced by local selection and detected clear geographical patterns of adaptive differentiation for both species. Adaptive differentiation within each species not only provides advanced information for designing functional units for conservation and management of the natural resources, but also helps predict the evolutionary trajectory of the populations against global climate change (Allendorf et al., 2010).

### 4.2 Linked selection before secondary contact shapes heterogeneous divergence

Pleistocene glacial fluctuations and the following postglacial recolonization have played critical but complicated roles in speciation or divergence, by repeated glacial isolation and mix (Duranton et al., 2018; Nevado et al., 2018; Wang et al., 2016). Looking into the demographic history is necessary to disentangle the factors that shape the patterns of both population differentiation and genome‐wide divergence (O'Connell et al., 2021). Herein, interspecific divergence could be best explained by the SC2N2mG scenario, where differential introgression and linked selection are suggested to have shaped the pattern of heterogeneous genomic divergence (Rougeux et al., 2017).

Under the SC2N2mG scenario, a majority of the genome was suggested to have reduced effective size compared to the rest of the genome of either or both species, although the degree to which the effective population size locally reduced is not very high (~7%). As expected under the scenario of linked selection, we observed both reduced genetic diversity and recombination rate in genomic islands in comparison to the rest of the genome, and significantly positive correlations between genetic diversity and recombination rate in both species, and a negative correlation between recombination rate and differentiation (*F*_ST_) (Burri et al., 2015; Cruickshank & Hahn, 2014; Duranton et al., 2018). Interestingly, we found a good conservation of both nucleotide diversity and recombination landscapes between species. Such shared patterns of genetic diversity strongly suggest that linked selection in the common ancestral lineage of the two studied species has shaped the observed heterogeneous genomic landscape between species. This is because recombination rate is the key mediator of linked selection, and linked selection based on either background selection or selective sweeps can reduce genetic variation in ancestral population and consequently generate shared patterns of reduced genetic diversity in regions of low recombination (Burri et al., 2015; Cruickshank & Hahn, 2014; Vijay et al., 2017). This result is similar to the observations in *Ficedula* flycatchers (Burri et al., 2015), Darwin's finches (Vijay et al., 2017) and *Primulina* species (Ke et al., 2022), where the shared patterns of heterogeneous genomic landscapes between species or lineages are contributed by linked selection in low recombination regions.

However, under the scenario of linked selection, *D*_xy_ is expected to be unchanged or reduced in highly differentiated regions due to the effects of recurrent background selection or selective sweeps, which reduce sequence diversity in these regions (Burri et al., 2015; Cruickshank & Hahn, 2014; Vijay et al., 2017). Contrary to this expectation, we observed elevated *D*_xy_ in genomic islands in comparison to the rest of the genome and a positive correlation between *D*_xy_ and *F*_ST_. These data suggest that additional evolutionary factors that can increase absolute divergence have probably remodelled the heterogeneous genomic divergence landscapes that have been previously shaped by linked selection in ancestral lineage. Under the scenarios of divergent sorting of ancient polymorphisms and speciation‐with‐gene‐flow or secondary contact, *D*_xy_ is expected to elevate in highly differentiated regions, leading to a positive correlation between *D*_xy_ and *F*_ST_ (Cruickshank & Hahn, 2014; Han et al., 2017; Ma et al., 2018). In particular, linked selection can accelerate lineage sorting in low recombination regions by increasing the chance to fix alternative alleles in these regions, generating heterogeneous genomic divergence between diverging populations at the initial stage of geographic isolation (Duranton et al., 2018). Divergent sorting of ancient haplotypes after the onset of speciation is common and predicted to elevate *D*_xy_ and have reduced lineage‐specific genetic measures (ρ, π and Tajima's *D*) in genomic islands (Han et al., 2017; Ke et al., 2022; Ma et al., 2018). Under this scenario, global patterns of positive correlations between *F*_ST_ and *D*_xy_, between genetic diversity and *D*_xy_, and between recombination rate and *D*_xy_, are also expected (Ke et al., 2022; Ma et al., 2018; Wang et al., 2019). Here, we observed the same global patterns of positive correlations for these genetic diversity measures in both species, suggesting that divergent sorting of ancient polymorphisms probably with the aid of linked selection, divergent local selection and genetic drift has also played important roles in promoting heterogeneous genomic divergence (Duranton et al., 2018; Han et al., 2017; Ma et al., 2018). In addition, recombination landscape per se can not only influence linked selection but also divergent sorting of ancient polymorphisms, leading to a similar pattern of *D*_xy_ as observed in this study (Duranton et al., 2018). Other than these factors, the differential introgression following secondary contact scenario as revealed by demographic modelling also generates the same pattern of *D*_xy_ in highly differentiated regions, where gene flow is restricted and thus absolute sequence divergence is elevated (Cruickshank & Hahn, 2014; Han et al., 2017; Ma et al., 2018).

### 4.3 Differential introgression following secondary contact promotes heterogeneous genomic divergence

The inferred demographic model suggests that differential introgression following secondary contact is also responsible for the heterogeneous genomic divergence. We found that both *F*_ST_ and *D*_xy_ were increased, while all lineage‐specific measures (ρ, π and Tajima's *D*) were reduced in genomic islands, which are in accordance with the secondary contact scenario (Burri et al., 2015; Cruickshank & Hahn, 2014; Duranton et al., 2018). Under this scenario, the split between the two sister species was estimated to be ~1.2 Ma, overlapping with the Calabrian stage of Pleistocene, when recurrent divergence events of marine animals were revealed to occur in the West Pacific (Jiang et al., 2021; Shahdadi et al., 2022; Zhai et al., 2012). The following postglacial contact was suggested to start recently at ~0.8 Ma. This implies that *L. polyactis* and *L. crocea* had evolved in allopatry for ~0.4 million years. The estimation of the time of the split between species was noticeably more recent than that reported in a previous study (Liu et al., 2010). This is probably due to the estimation taking the demographic models with heterogeneous population size and migration rates into consideration (Guirao‐Rico et al., 2017; Liu et al., 2020). It is also probably due to the underestimation of generation time, which can be longer in the past colder environment.

Under the best supported model, we observed reduced gene flow in genomic islands by more than one order of magnitude in comparison to the rest of the genome. It has been shown that heterogeneous migration rates are common at intermediate stages of speciation in various animal and plant systems (Liu et al., 2020; Rougeux et al., 2017; Tine et al., 2014). The overall effective gene flow throughout the genome islands was estimated to not be enough to counteract the effects of genetic drift (*N*_m_ <1). In contrast, gene flow estimated based on the genome that is free of genomic islands was effective to counteract the effects of genetic drift (*N*_m_ >1). These data suggest that genetic barriers to gene flow could have been established in genomic islands (Duranton et al., 2018; Wang et al., 2016). Upon secondary contact following glacial retreat, genetic differentiation in regions that are not related to adaptive differentiation or reproductive isolation was eroded by gene flow (Cruickshank & Hahn, 2014; Harrison & Larson, 2016). Therefore, restricted gene flow has resulted in highly differentiated regions and elevated *D*_xy_ in these regions in comparison to the rest of the genome.

The rising of genomic islands can be strengthened through postzygotic isolation mechanisms after secondary contact, when intermediate hybrids are selected against (Butlin & Smadja, 2018; Duranton et al., 2018). This mechanism can further strengthen the effect of genetic barriers to gene flow in genomic islands (Butlin & Smadja, 2018). In addition, chromosomal rearrangements can reduce gene flow and affect linked isolation genes by altering local recombination landscape, prompting the evolution of reproductive barriers (Crown et al., 2018; Rieseberg, 2001). Here, evidence for chromosomal inversions were observed to coincide with genomic islands between the two species, addressing the important roles of genomic islands in speciation in the studied system. Moreover, genomic islands generated by linked selection can be involved in reproductive isolation during early allopatric speciation, although some of them are incidental and can be eroded by gene flow (Duranton et al., 2018). Furthermore, highly differentiated regions generated due to divergent sorting of ancient polymorphisms during the early divergence period, can also act as barriers to gene flow upon secondary contact (Duranton et al., 2018). Finally, we found reduced recombination rate in genomic islands compared to the rest of the genome. Low recombination rate helps cumulating the effect of multiple mutations contributing to reproductive isolation, such that they resist more to gene flow when linked together on a same haplotype (Duranton et al., 2018). In mouse and butterflies, genetic barriers to introgression are suggested to be shaped by recombination rate variation, with a positive correlation between introgression and recombination rate (Janoušek et al., 2015; Martin et al., 2019). Above all, differential gene flow is a major factor promoting genomic islands of interspecific divergence in the second phase of divergence under secondary contact scenario. The reduced introgression in low‐recombining regions is probably related to the effects of linked selection, divergent sorting of ancient polymorphisms, genetic drift, accumulation of different mutations and selection against introgressed alleles from the alternated species due to for example, genetic incompatibilities (Jahner et al., 2021; Martin & Jiggins, 2017; Pazhenkova & Lukhtanov, 2021).

### 4.4 Lack of correlated pattern between interspecific and intraspecific divergence

Previous studies in birds have shown that the levels of genetic diversity and differentiation are highly correlated among population‐, species‐ and even higher‐order taxa (Burri et al., 2015; Van Doren et al., 2017; Vijay et al., 2017). It is suggested that similar processes (e.g., linked selection in specific genomic regions of low recombination) have probably been underlying such genome‐wide heterogeneous landscapes of divergence (Burri et al., 2015; Van Doren et al., 2017; Vijay et al., 2017). In this study, we first observed both conserved nucleotide diversity and in particular recombination landscapes between the two species, implying correlated variations in the intensity of linked selection between intraspecific and interspecific genetic diversity. However, we found that recombination rates were reduced in intraspecific genomic islands in *L. polyactis* but not in *L. crocea*. In particular, ~8% of intraspecific genomic islands were revealed to overlap between the two species, and ~5% interspecific genomic islands were overlapping with those of intraspecific divergence. However, these ratios of overlapping between interspecific and intraspecific genomic islands are lower than those of the expectation under random distribution. These data show little support to the hypothesis that linked selection in ancient lineage of the two diverging species and local introgression of divergent alleles from the alternated species, respectively, have generated similar patterns of genomic islands for intraspecific divergence and between intraspecific and interspecific divergence. One explanation is that high gene flow within each species has probably overwhelmed the effect of linked selection and low recombination rate, and eroded the highly differentiated regions. It is also possible that incomplete lineage sorting at the early stage of speciation between the two diverging species has reshaped the genome‐wide landscapes of divergence within each species (Hobolth et al., 2011). We can also not exclude the possibility that divergent alleles that are locally introgressed from alternated species are so strongly selected against by natural selection that they are eliminated from each species. The slight overlapping of intraspecific genomic islands might be due to local selection from similar selective agents, although this would require continuous or frequent occurrences of selective sweeps at the same genomic regions between species (Vijay et al., 2017). Taken into consideration the overlapping of their distribution ranges, it is likely that the two species have evolved in parallelism under similar selective pressure, which explains the slight overlapping of intraspecific genomic islands.

## 5 CONCLUSIONS

Integration of genome‐wide variations with demographic history pave a way to disentangle the evolutionary processes shaping heterogeneous genome‐wide divergence. We observed both extensive interspecific and intraspecific genomic islands across the studied species. While a proportion of the genomic islands have evolved due to linked selection shaped by variation in recombination rate across the genome before secondary contact, such pattern of heterogeneous genomic divergence has been reshaped by differential introgression following secondary contact. Discrimination of the factors underlying these genomic islands would help understand the genomic basis of speciation in organisms.

## AUTHOR CONTRIBUTIONS

Le Wang and Zining Meng conceived and designed the study. Zining Meng supervised the whole study. Shufang Liu, Zhimeng Zhuang and Le Wang collected samples. Zining Meng, Shufang Liu, Le Wang and Yang Yang performed genome sequencing, RNA sequencing and RAD sequencing. Le Wang, Zining Meng, and Yang Yang annotated the genome and analysed the data. Le Wang, Zining Meng and Shufang Liu wrote the manuscript with input from the other authors. All authors discussed the results and approved the final version of the study.

## CONFLICT OF INTEREST

The authors declare no conflict of interest.

## Supporting information


Figure S1

Table S1

Appendix S1

## Data Availability

The genome sequences and all raw sequencing reads for this study have been archived in the National Genomics Data Center of China (NGDC) and China National Gene Bank (CNGB) database with Bioproject accession numbers of PRJCA003706 and CNP0001482, respectively. Genome sequences and SNP datasets would be archived in the Dryad Digital Repository (https://doi.org/10.5061/dryad.kwh70rz6j).
