# Supplementary material for: Linked selection, differential introgression and recombination rate variation promote heterogeneous divergence in a pair of yellow croakers
Source: Mol Ecol. 2022 Sep 29;31(22):5729–44. doi: 10.1111/mec.16693 (PMC9828471; doi:10.1111/mec.16693)
Supplement: Supplementary file 1 — Figure S1 [file MEC-31-5729-s001.docx]

**Supplementary Figures**


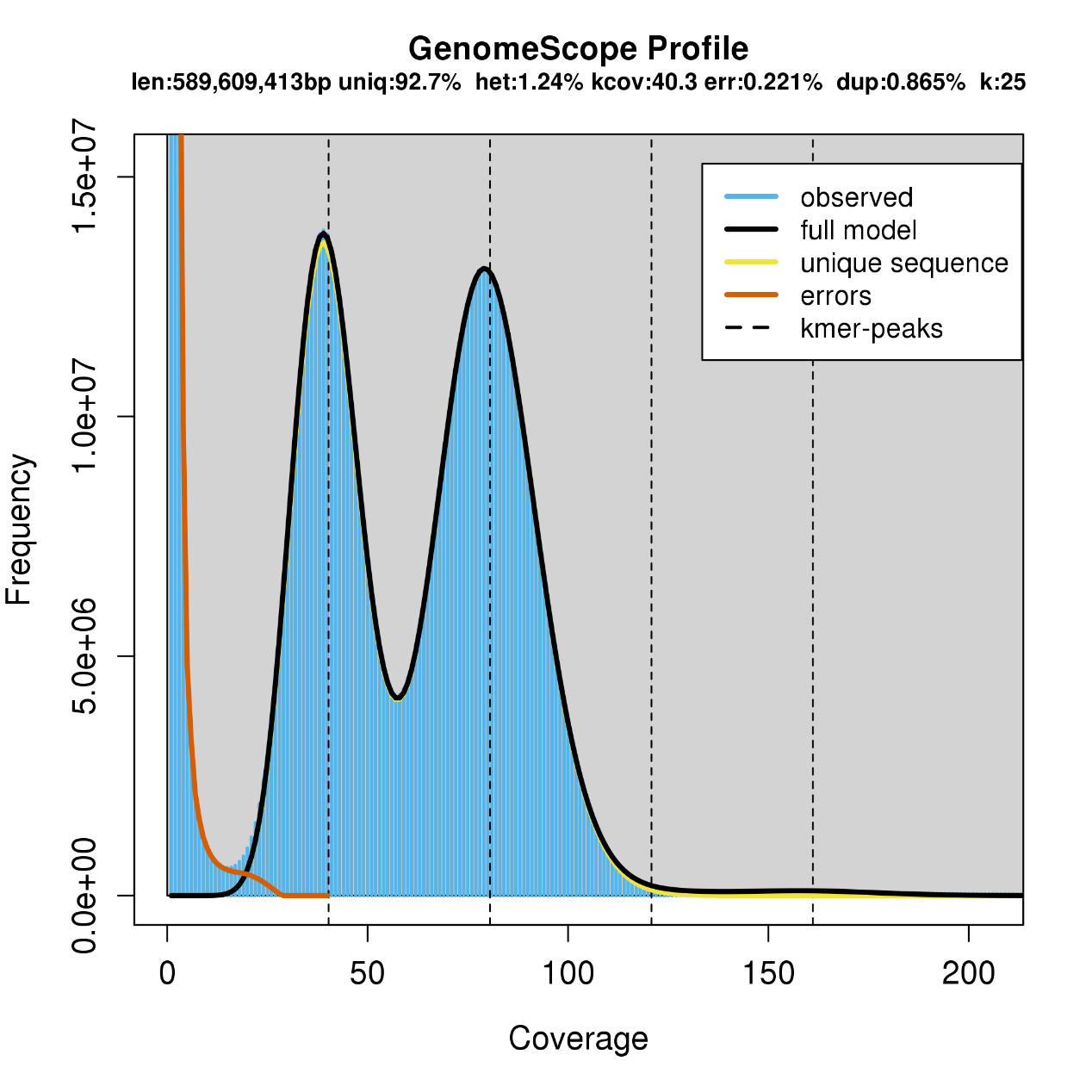


**Figure S1** Distribution of k-mer depth in *L. polyactis* estimated based on Illumina paired-end and mate-paired sequencing reads using the program Allpaths-LG.


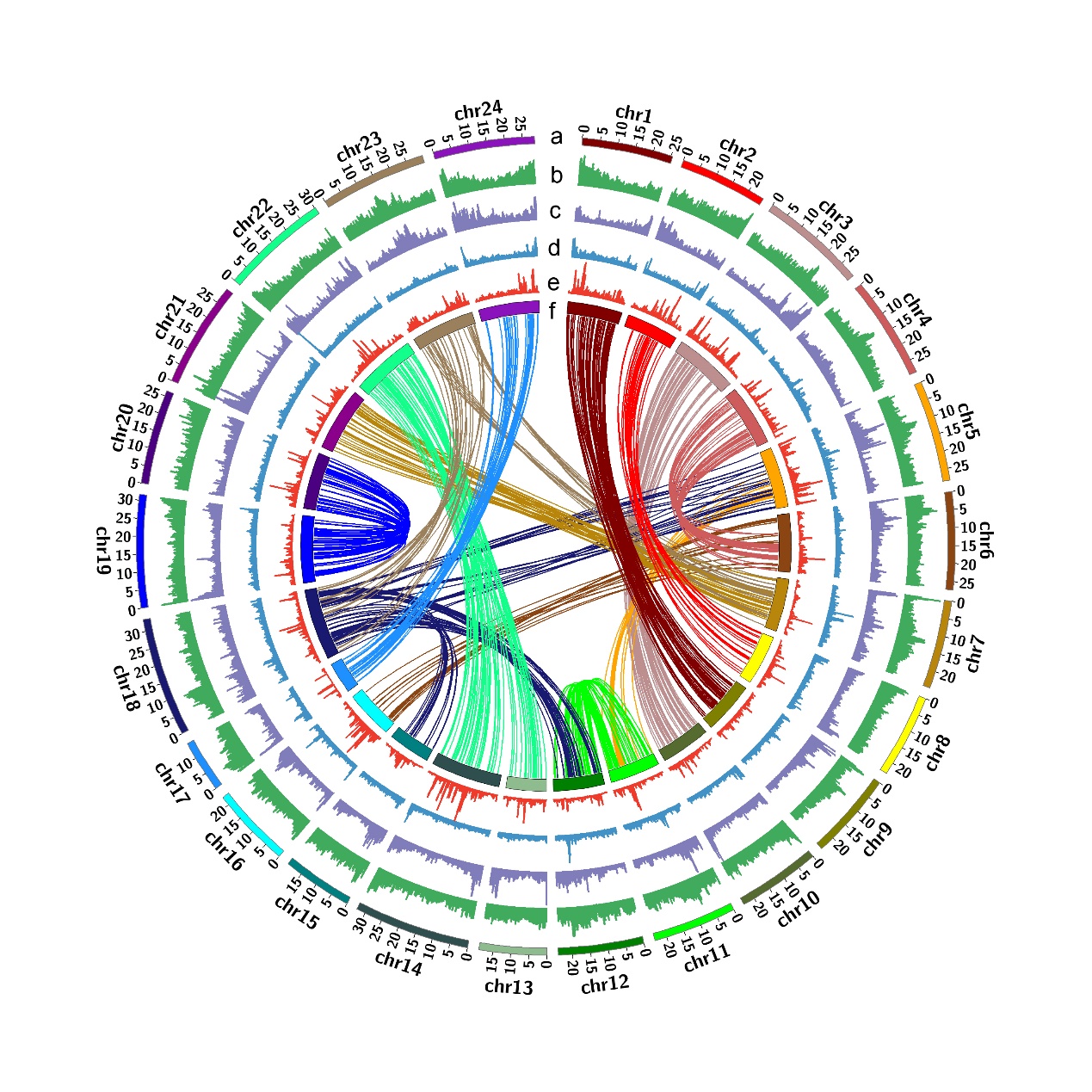


**Figure S2** Circos plot of annotated genomic elements *in L. polyactis* genome. From outer to inner layers showing (**a**) pseudochromosomes, (**b**) distribution of GC content, (**c**) repeated sequences, (**d**) recombination rates, (**e**) conserved noncoding elements (CNEs) in 500-kb windows, and (**f**) collinearity between chromosomes of *L. polyactis*, showing historical chromosome duplications.

**Figure S3** Pearson’s correlations between annotated genomic elements in *L. polyactis*. Pearson’s correlation coefficient (*R*) and significance (*P*) were shown between GC content and average recombination, Rho/ρ (a), between GC content and content of sequence repeats (b), between GC content and content of CNEs (c), between average reombination rate (Rho/ρ) and content of sequence repeats (d), between average reombination rate (Rho/ρ) and content of CNEs (e) and between content of sequence repeats and content of CNEs (f), estimated with a 500-kb window size. (g) heatmap of correlations among GC content, repeat sequences, recombination rates and concentration of CNEs.


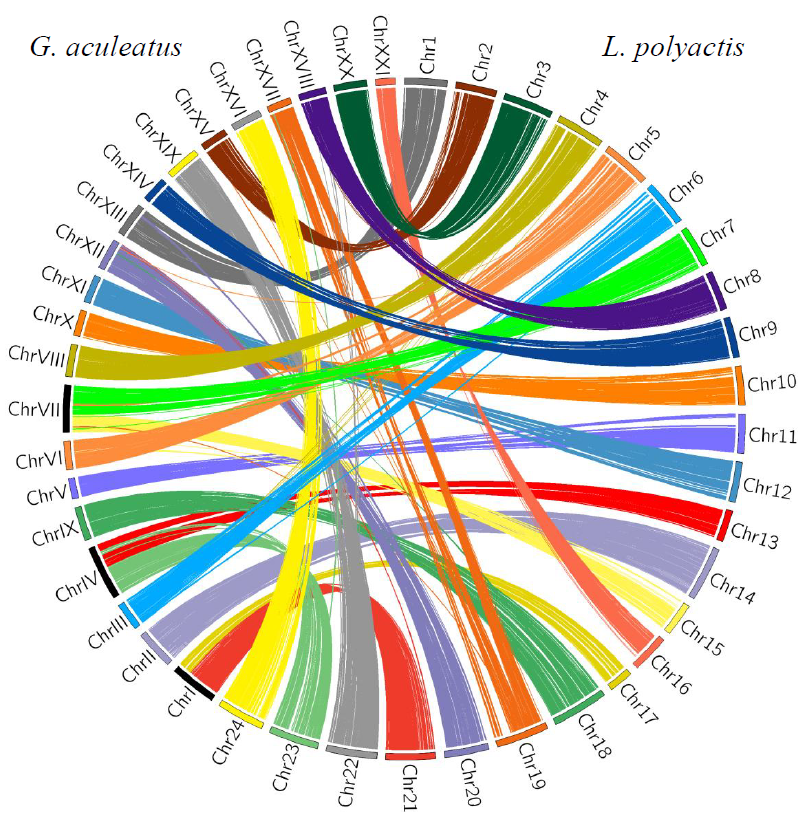


**Figure S4** Circos plot of genomic synteny between stickleback (*G. aculeatus*, 21 pairs of chromosomes) and small yellow croaker (*L. polyactis,* 24 pairs of chromosomes). Chromosome split events were observed at three chromosomes of stickleback (ChrI, ChrIV and ChrVII), highlighted with black color.


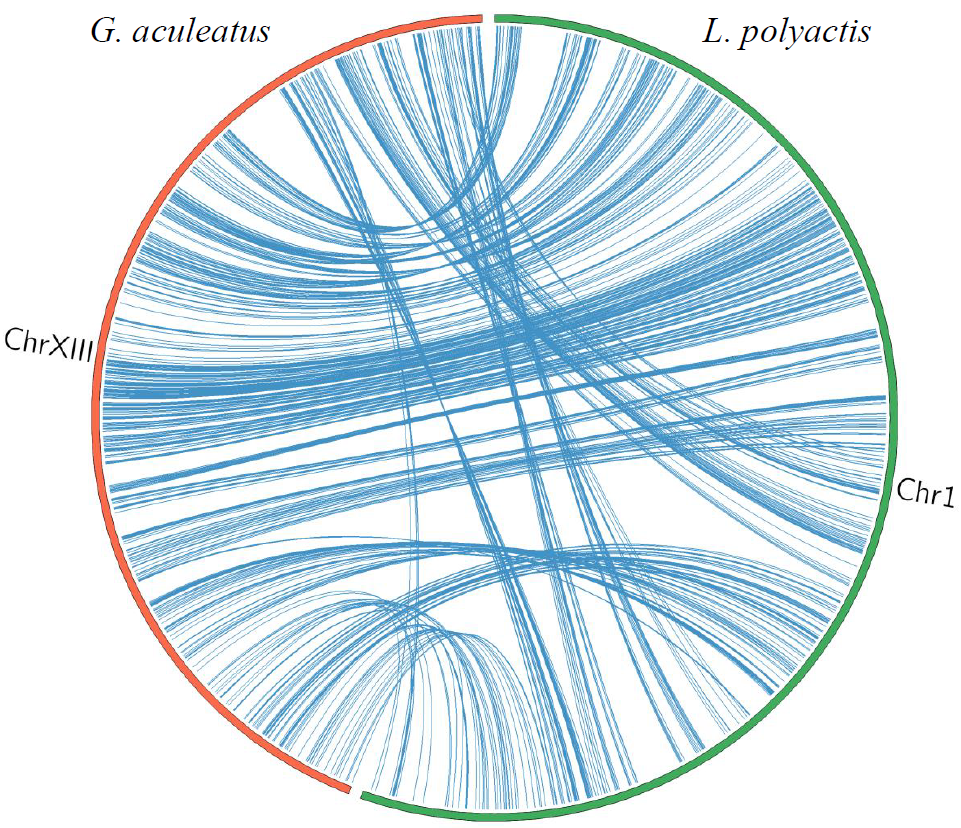


**Figure S5** Circos plot of genomic synteny between *G. aculeatus* and *L. polyactis*, showing detailed syntenic relationship between genomic fragments of these two species, within a pair of chromosomes (Chr1 of *L. polyactis* vs ChrXIII of *G. aculeatus*), where evidence of genome rearrangement was observed.


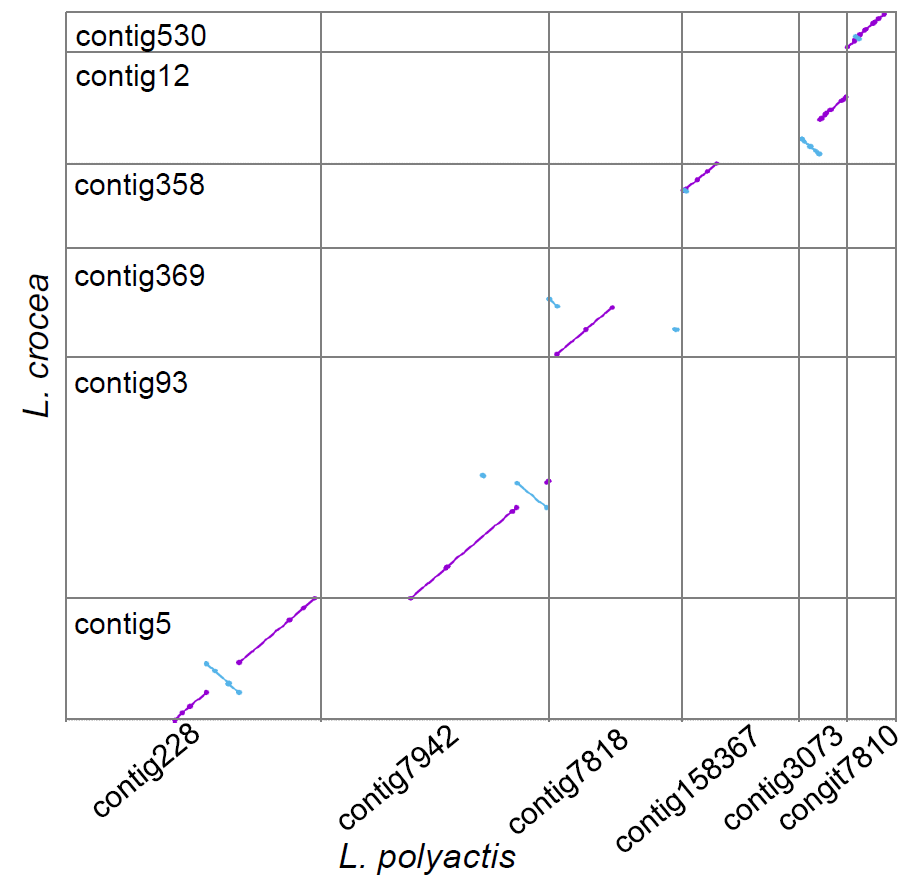


**Figure S6** Oxford plot of genome synteny between six pairs of contigs, showing chromosome inversions (with a length of 2.1Mb, 1.7Mb, 230kb, 16kb, 490kb and 11kb, respectively) between the two sister species, *L. polyactis* and *L. crocea*.


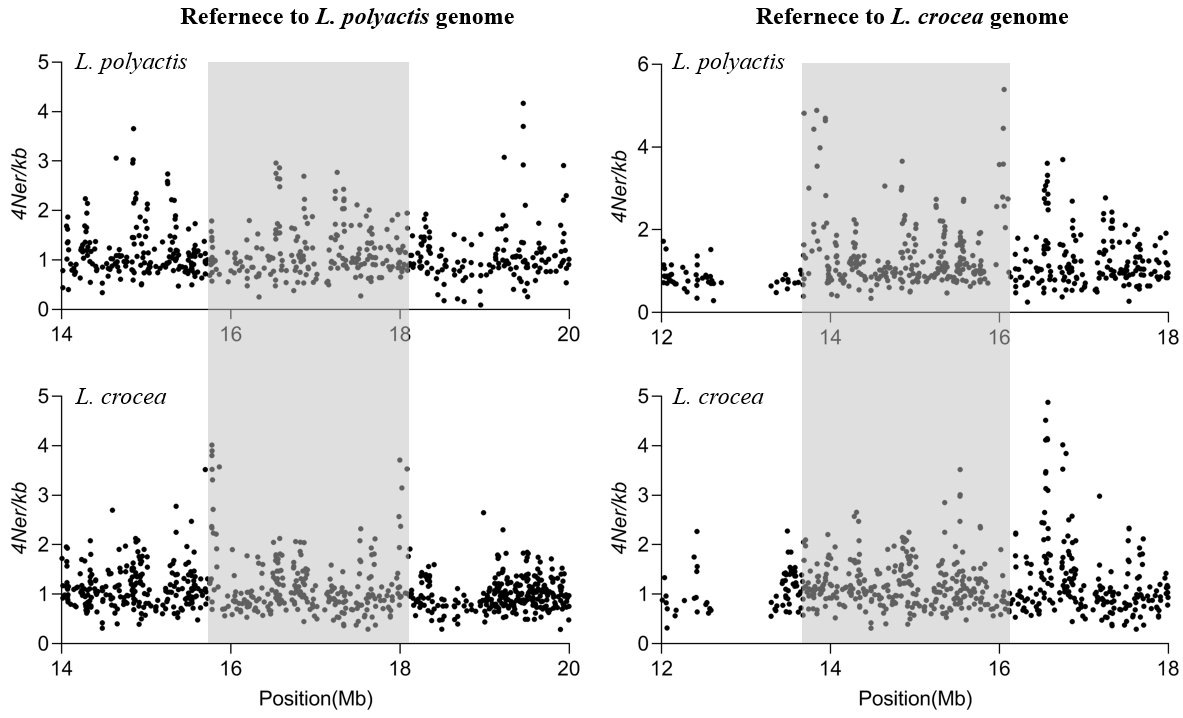


**Figure S7** Verification of the largest chromosome inversion (~ 2Mb) in contig228 between *L. polyactis* and *L. crocea*, as shown in **Figure S6**, using chromosome-wide recombination rates (rho = 4Ner/kb), estimated based on RADSeq derived population data. When chromosome-wide recombination rates for the two sister species were estimated using *L. polyactis* genome as reference, elevated recombination rates at both edges of the inverted fragment were observed in *L. crocea*, compared to the reference species, *L. polyactis*, where showed background-level recombination rates (as shown in the shading area). When recombination rates were estimated using *L. crocea* genome as reference, elevated recombination rates at both edges of the inverted fragment were observed in *L. polyactis*, compared to the reference species.


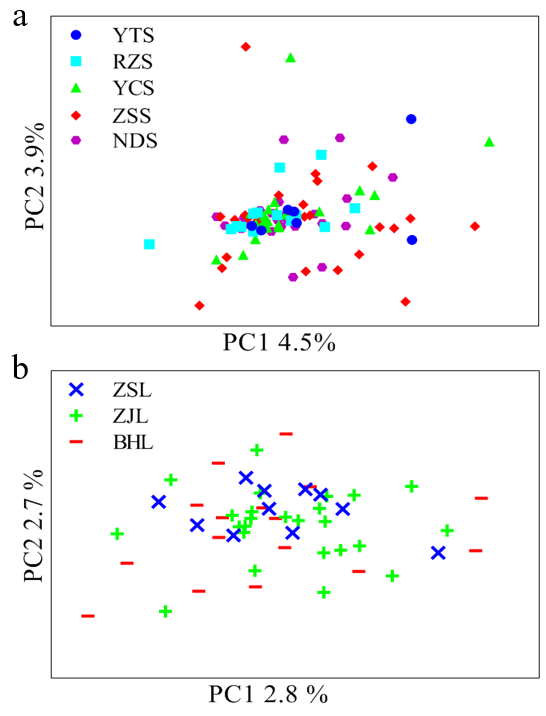


**Figure S8** Principal component analysis of genetic relationships among individuals within *L. polyactis* (a) and *L. crocea* (b).


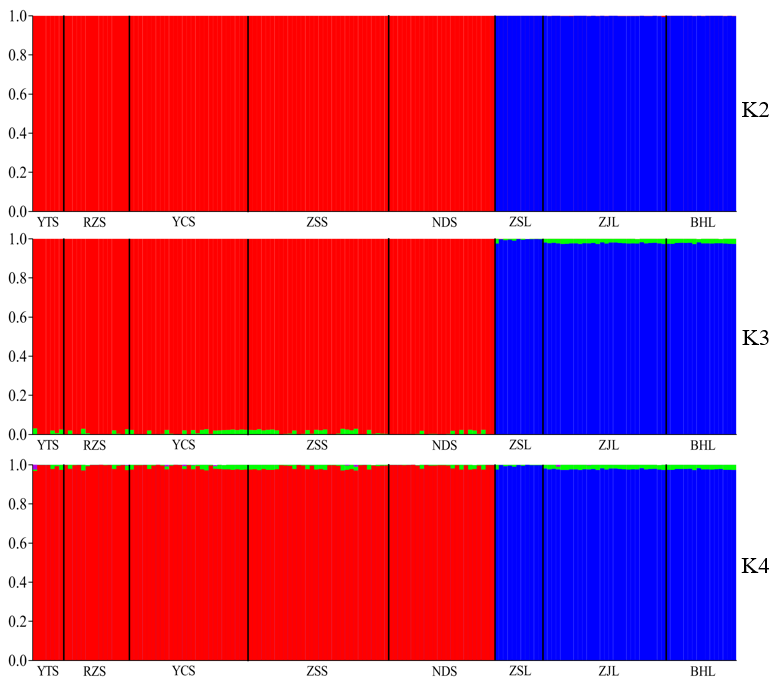


**Figure S9** Genetic clusters among individuals between the two sister species *L. polyactis* and *L. crocea* at K=2, 3 and 4, as revealed by the program Admixture, based on common SNPs.


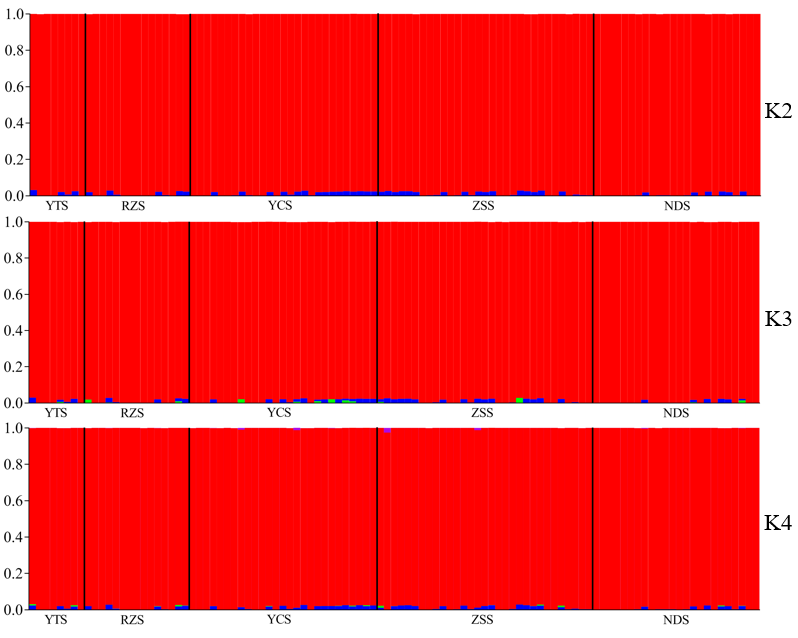


**Figure S10** Genetic clusters among individuals of five locations of *L. polyactis* at K=2, 3 and 4, as revealed by the program Admixture.


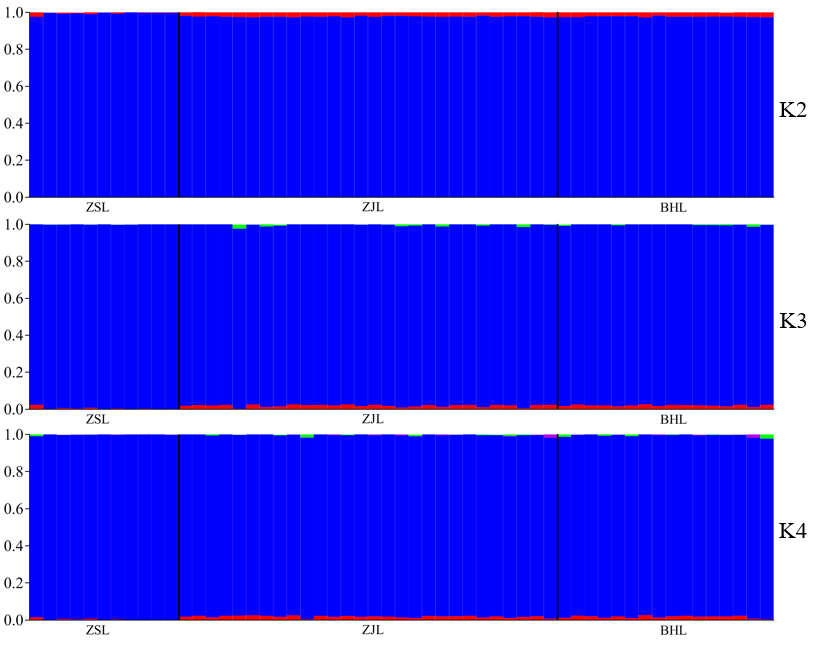


**Figure S11** Genetic clusters among individuals of three locations *L. crocea* at K=2, 3 and 4, as revealed by the program Admixture.


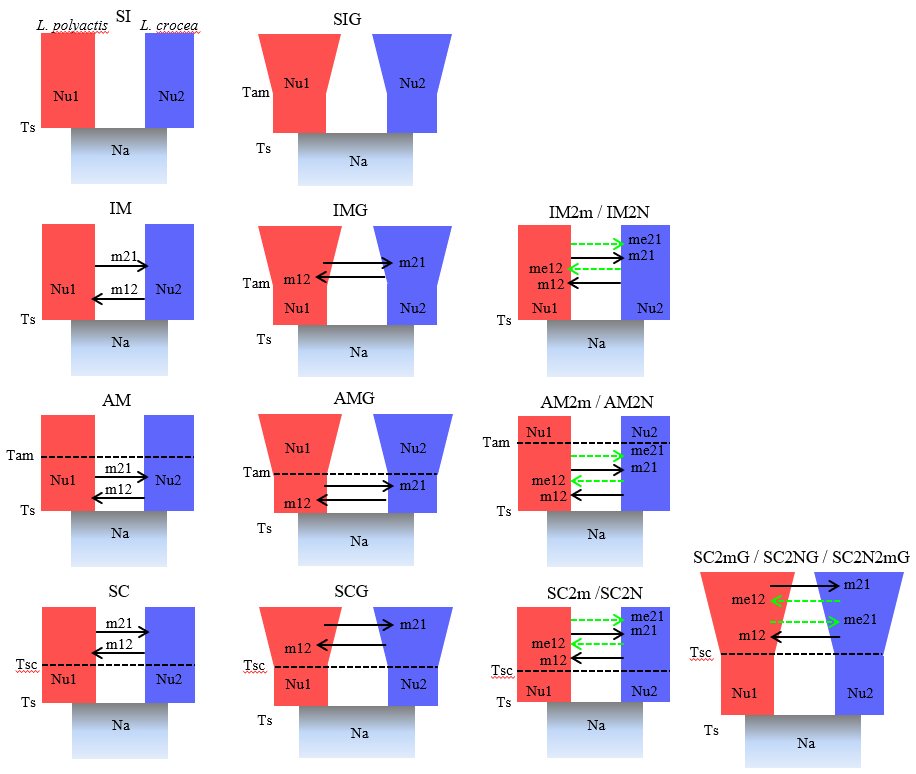


**Figure S12** Demographic models used to fit the divergence between *L. polyactis* and *L. crocea*. Four standard demographic models of historical divergence include the Strict Isolation (SI), Isolation-with-Migration (IM), Ancient Migration (AM) and Secondary Contact (SC), where allows for one shared migration rate in each direction (m12, m21) across the genome (homogeneous migration). Four modified models of SI, IM, AM and SC that were named with SIG, IMG, AMG and SCG, respectively, allow for population growth. Three modified models of IM, AM and SC that were named with IM2m, AM2m and SC2m, respectively, allow for heterogeneous migration of gene flow (me12 and me21). Three modified models of IM, AM and SC that were named with IM2N, AM2N and SC2N, considers the variation in effective population size due to the effect of linked selection by considering two categories of loci in the genome (Hill-Robertson effects). The best fit models (SC/SCG/SC2m/SC2N) in each category were further optimized to fit the frequency spectrum of SNP data set, by allowing for population growth, heterogeneous migration and/or Hill-Robertson effects (SC2mG, SC2NG and SC2N2mG).


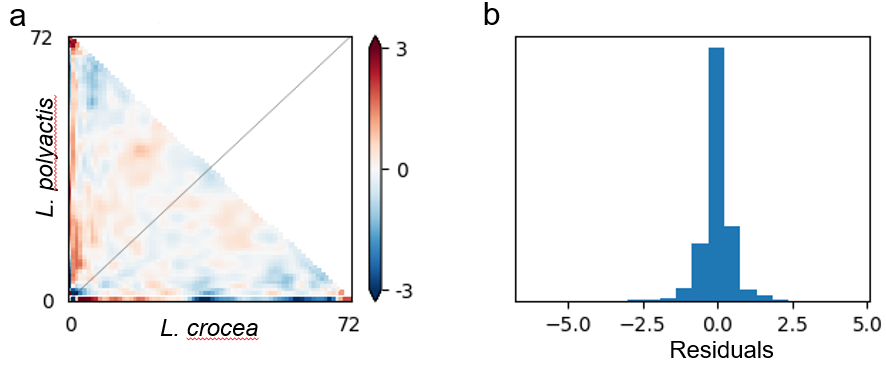


**Figure S13** Residuals of the best fit model of the divergence (SC2N2mG) between *L. polyactis* and *L. crocea*. a and b showed the heat map and frequency distribution of spectrum that is not captured by the best fit model, where red and blue indicate too many and too few SNPs that deviate from the expected scenario, respectively.


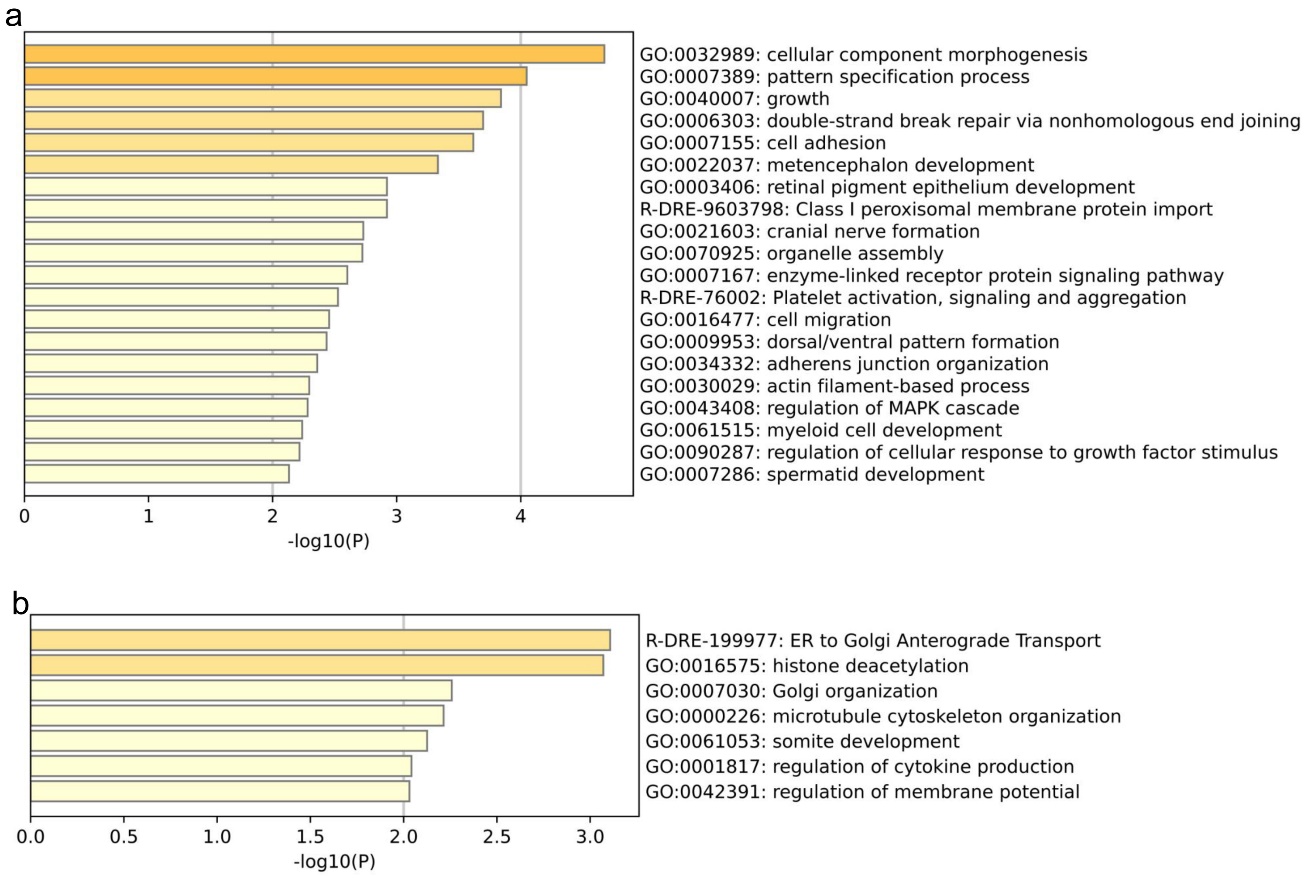


**Figure S14** Gene ontology analysis of genes under putative local selection in *L*. *polyactis* (a) and *L. crocea* (b).


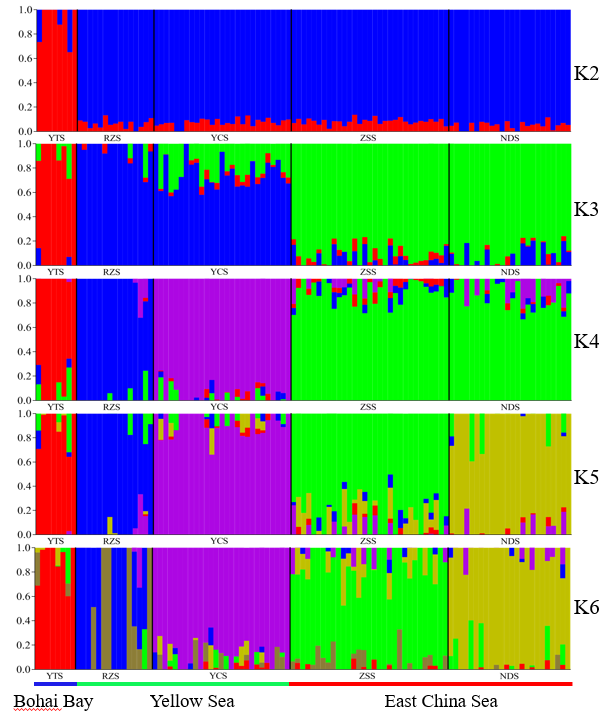


**Figure S15** Genetic clusters among individuals of five locations of *L. polyactis* at K=2, 3, 4, 5 and 6, based on species-specific outlier loci, where samples were differentiated with each other according to their geographical locations at the most likely K value of 5.


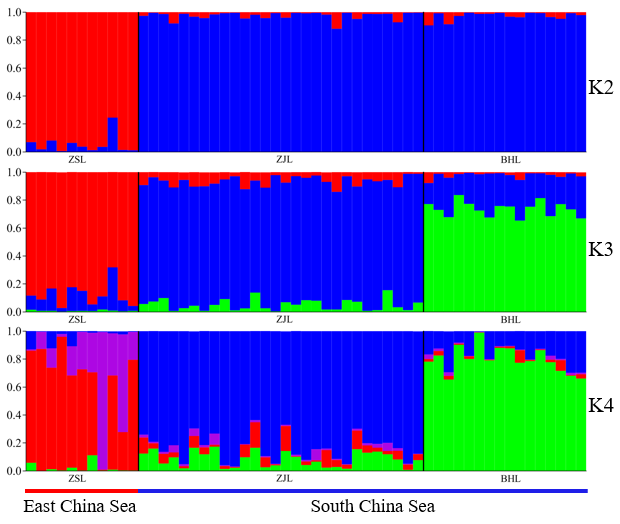


**Figure S16** Genetic clusters among individuals of three locations of *L. crocea* at K=2, 3 and 4 based on species-specific outlier loci, where samples were differentiated with each other according to their geographical locations at the most likely K value of 3.


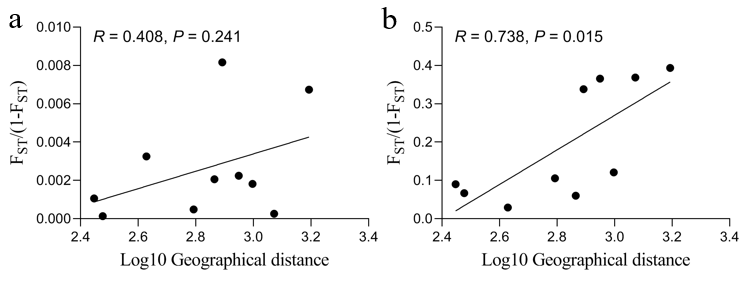


**Figure S17** Isolation-by-distance examined using Mantel tests based on putative neutral SNPs (a) and outliers (b).


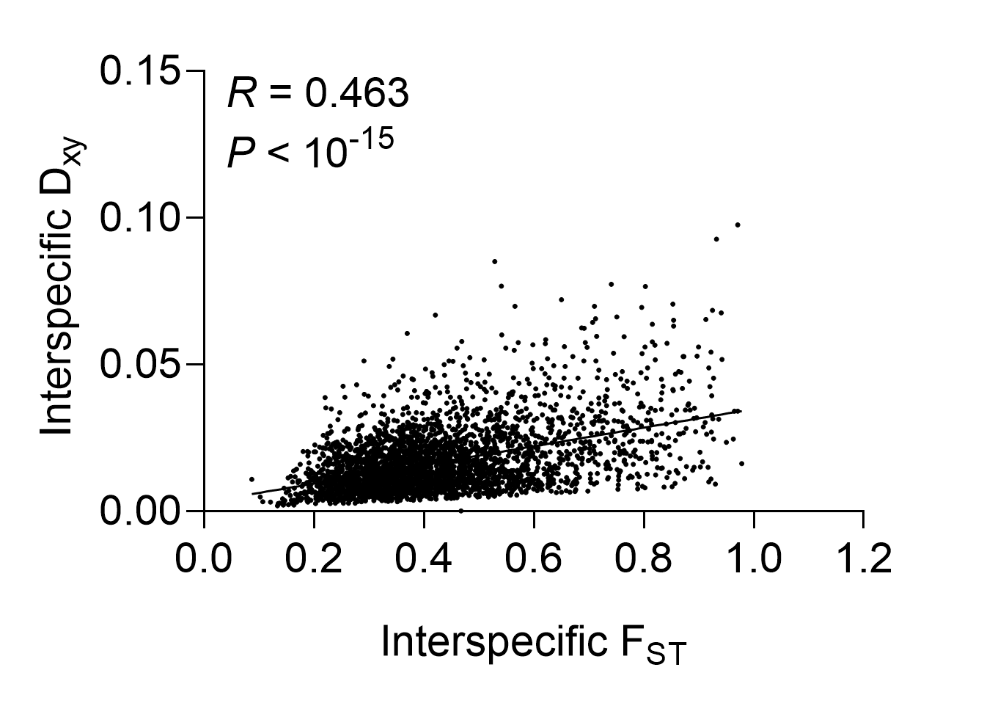


**Figure S18** Pearson’s correlation of interspecific divergence between interspecific *F*_ST_ and *D*_xy_ between *L. polyacits* and *L. crocea*.


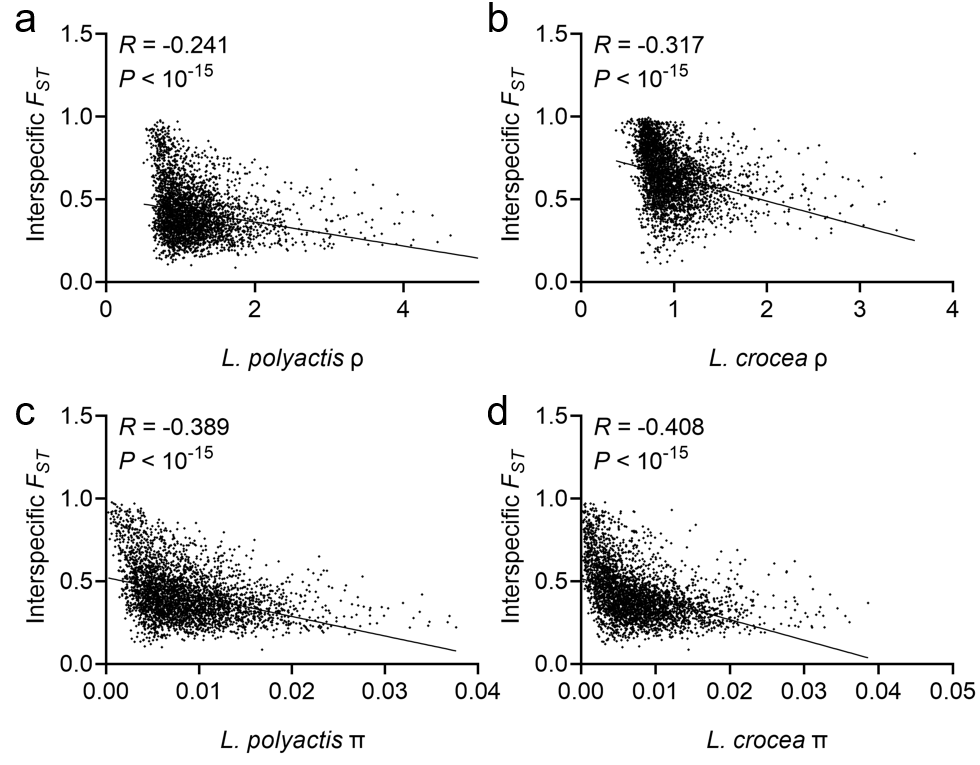


**Figure S19** Pearson’s correlation between recombination rates (ρ) and interspecific *F*_ST_ separately in *L. polyactis* (a) and *L. crocea* (b), and between nucleotide diversity (π) and inter-specific *F*_ST_ separately in *L. polyactis* (c) and *L. crocea* (d).
