# Supplementary material for: Linked selection, differential introgression and recombination rate variation promote heterogeneous divergence in a pair of yellow croakers
Source: Mol Ecol. 2022 Sep 29;31(22):5729–44. doi: 10.1111/mec.16693 (PMC9828471; doi:10.1111/mec.16693)
Supplement: Supplementary file 3 — Appendix S1 [file MEC-31-5729-s002.docx]

**Supplementary Note**

**Genome annotation**

Sequences were firstly softmasked using RepeatMasker v4.0.7 (Chen, 2004) based on the repeat libraries obtained by running RepeatModeler v1.0.11 (http://www.repeatmasker.org) and Repbase database (Jurka et al., 2005). The mRNA sequencing reads were cleaned as described above and assembled using Trinity v2.2.0 (Grabherr et al., 2011) with default parameters. Assembled transcripts and protein sequences of zebrafish, medaka, stickleback, fugu and Nile tilapia (Ensembl release 96) were mapped to the genome and used as hints for evidence-based prediction of gene models. SNAP v2013 (Korf, 2004) and Augustus v3.2.3 (Stanke & Morgenstern, 2005) were iteratively trained for three times to improve the prediction of gene models. Predicted gene models were further annotated by blastp to nr database. We further predicted conserved non-coding elements (CNEs), according to a previous method (Brawand et al., 2014). Pairwise whole-genome alignment was carried out between *L. polyactis* and each of the following fish species: zebrafish, medaka, stickleback, fugu and Nile tilapia (Ensembl database, release 96), with the program LASTZ v1.04 (Harris, 2007). Conserved sequences in at least one pair of alignments were extracted and reduced to single-coverage with respect to the reference genome of *L. polyactis*. These single-coverage sequences were used as targets for multiple alignment. The program PhastCons (Siepel et al., 2005) was used for prediction of CNEs with the parameters: target coverage of input alignments ≥ 0.3 and average conserved sequence length ≥ 45 bp. Predicted CNEs were then filtered by aligning to coding sequences, transcripts, non-coding RNAs, pseudogenes and transposable elements of the studied species with an E-value cutoff < 1E-10. Only elements of > 30 bp in length and of repetitive content < 50% were considered as putative CNEs.

**References**

Brawand, David, Wagner, Catherine E, Li, Yang I, Malinsky, Milan, Keller, Irene, Fan, Shaohua, et al. (2014). The genomic substrate for adaptive radiation in African cichlid fish. *Nature, 513*(7518), 375-381.

Chen, Nansheng. (2004). Using Repeat Masker to identify repetitive elements in genomic sequences. *Current Protocols in Bioinformatics, 5*(1), 4.10. 11-14.10. 14.

Grabherr, Manfred G, Haas, Brian J, Yassour, Moran, Levin, Joshua Z, Thompson, Dawn A, Amit, Ido, et al. (2011). Trinity: reconstructing a full-length transcriptome without a genome from RNA-Seq data. *Nature Biotechnology, 29*(7), 644.

Harris, Robert S. (2007). Improved pairwise Alignmnet of genomic DNA. *Ph.D. Thesis, The Pennsylvania State University*.

Jurka, Jerzy, Kapitonov, Vladimir V, Pavlicek, A, Klonowski, P, Kohany, O, & Walichiewicz, J. (2005). Repbase Update, a database of eukaryotic repetitive elements. *Cytogenetic and Genome Research, 110*(1-4), 462-467.

Korf, Ian. (2004). Gene finding in novel genomes. *BMC Bioinformatics, 5*(1), 59.

Siepel, Adam, Bejerano, Gill, Pedersen, Jakob S, Hinrichs, Angie S, Hou, Minmei, Rosenbloom, Kate, et al. (2005). Evolutionarily conserved elements in vertebrate, insect, worm, and yeast genomes. *Genome Research, 15*(8), 1034-1050.

Stanke, Mario, & Morgenstern, Burkhard. (2005). AUGUSTUS: a web server for gene prediction in eukaryotes that allows user-defined constraints. *Nucleic Acids Research, 33*(suppl_2), W465-W467.
